# Design Considerations, Formulation Approaches, and Strategic Advances of Hydrogel Platforms for Tendinopathy Management

**DOI:** 10.34133/bmr.0299

**Published:** 2026-01-29

**Authors:** Junhao Lin, Xuan Yao, Hongyan Zhou, Yuheng Li, Jie Liao, Shiwu Dong, Wenhui Hu

**Affiliations:** ^1^ Department of Biomedical Materials Science, College of Biomedical Engineering, Third Military Medical University, Chongqing 400038, P.R. China.; ^2^ Department of Clinical Hematology Faculty of Laboratory Medicine, Third Military Medical University, Chongqing 400038, P.R. China.; ^3^ State Key Laboratory of Trauma and Chemical Poisoning, Third Military Medical University, Chongqing 400038, P.R. China.; ^4^ Department of Basic Medicine, Frontier Medical Service Training Brigade, Third Military Medical University, Changji, Xinjiang 831200, P.R. China.

## Abstract

Tendinopathy is a musculoskeletal disorder characterized by severe pain that may persist for weeks or months, often resulting in disability. Existing treatments primarily consist of conservative interventions, including rest, nonsteroidal anti-inflammatory medications, localized corticosteroid injections, ultrasound, bracing, and stem cell-based therapies, as well as surgical procedures. However, therapeutic outcomes remain unsatisfactory. Consequently, there is an urgent need for effective strategies in tendinopathy management. As a bioengineered material, the hydrogel has been extensively studied for the treatment of tendinopathy due to its stable physicochemical properties, biocompatibility, degradability, mechanical robustness, injectability, and stimuli-responsive drug delivery capability. Based on the anatomical structure of tendons and therapeutic requirements during disease progression, hydrogels can be designed into various formulations, such as scaffolds, patches, sprays, microspheres, and injectable systems, depending on the raw materials, crosslinking methods, sizes, and morphological configuration. This review provides a comprehensive overview of the pathophysiological process involved in tendon healing and summarizes the considerations in the design of hydrogels in tendinopathy treatment. It emphasizes the therapeutic applications and stimuli-responsive properties of various hydrogel formulations in tendinopathy treatment, advancing the understanding of hydrogel-based strategies for tendinopathy management and focusing on formulation design. Additionally, the opportunities artificial intelligence brings to hydrogel research in design, optimization, and application advancement are also comprehensively discussed. Understanding the advances associated with hydrogel development is crucial for tendinopathy treatment.

## Introduction

Tendinopathy is a globally substantial health issue that profoundly affects individual well-being and healthcare systems worldwide [1,2]. Epidemiological data reveal that more than 30 million people experience tendon injuries annually, resulting in medical costs exceeding 140 billion. As life expectancy increases and age-related degeneration becomes more prevalent, approximately 25% of adults are expected to develop tendon disorders [3], with treatment costs ranging from $13,000 to 20,000 per case, compounded by extended treatment durations. Patients often experience impairments in quality of life due to chronic tendon pain, degenerative alterations, and potential fracture complications [4]. Tendinopathy typically arises from overuse or sudden compressive loading of the tendon. Clinical and animal studies have identified characteristic histopathological changes associated with tendinopathy, including collagen fiber degeneration, tissue disorganization, increased cellularity, and mild inflammatory responses. Furthermore, research has revealed that tendinopathic changes increase the risk of full-thickness tendon tears when subjected to higher mechanical loads [5]. Multiple contributing factors influence the development of tendinopathy. Intrinsic factors include age, gender, anatomical variations, body weight, and systemic diseases, whereas extrinsic factors involve sports activities, physical loadings, occupational demands, and environmental conditions such as walking surfaces and footwear [6]. Pathologically, tendinopathy is classified as either acute or chronic based on the disease course and is further subdivided into tendinitis, such as Achilles tendinitis [7] and tendinosis, such as peroneal tendinosis [8]. Tendinitis is characterized by inflammatory infiltration of the affected tendon and is commonly used to describe specific clinical syndromes. In contrast, tendinosis may remain asymptomatic and is considered a degenerative rather than an inflammatory condition [9].

Tendon injury repair is widely recognized to progress through 3 overlapping phases: inflammatory, proliferative, and remodeling. Each phase is characterized by distinct cellular activities and cytokine involvement while maintaining temporal overlap. The recovery period for tendinopathy is frequently prolonged and often yields suboptimal outcomes, partially due to the tendon’s intrinsically hypocellular and hypovascular nature, which compromises its healing capacity. Furthermore, a limited understanding of the underlying cellular and molecular mechanisms in tendinopathy has substantially hindered the development of novel therapeutic strategies, rendering tendon regeneration within musculoskeletal disorders a persistent clinical challenge [10]. Selecting clinical treatment strategies for tendinopathy is influenced by factors such as disease severity, anatomical location, and patient age. Conservative management is typically employed for partial tendon tears with preserved peritendinous vascularization. It may include rest, nonsteroidal anti-inflammatory drugs, local corticosteroid injections, immobilization, laser therapy, ultrasound, orthoses, growth factors, and stem cell therapy. In contrast, surgical intervention is typically reserved for complete tendon ruptures and primarily involves peripheral suturing to reconnect torn tissues [11]. However, current evidence indicates that existing therapies often fail to achieve complete functional tendon restoration. Conservative treatments are limited by poor healing capacity, scar formation, and the risk of rerupture. Similarly, surgical approaches are associated with complications such as scar tissue formation, postoperative infections, adhesions, and prolonged recovery times, frequently necessitating secondary procedures [12].

Recent studies have highlighted advancements using bioengineered hydrogels for treating tendinopathy. Hydrogels are 3-dimensional (3D) polymer networks composed of hydrophilic components containing polar functional groups and characterized by high water content. These networks are formed from crosslinked polymers connected through covalent bonds or noncovalent interactions, and their structures can be modified to accommodate various biomedical applications. Hydrogels possess stable physicochemical properties and soft, moist, and biocompatible characteristics, making them suitable as matrix components for engineering living cells [13]. Moreover, the porous structure of hydrogels facilitates the transport of drugs, growth factors, and cells, thereby establishing them as a promising platform for delivering pharmaceuticals or biomolecules. The drug delivery rate and targeting capability can be controlled by modifying the network structure and the interactions between drugs and polymer chains [14]. For instance, a hydrogel can down-regulate the nuclear factor-kappa B (NF-κB) pathway to regulate macrophage M2 polarization and efferocytosis, thereby maintaining immune homeostasis. It can also improve the inflammatory microenvironment, enhancing the clonogenicity, migration, and tenogenic differentiation capabilities of tendon stem/progenitor cells (TSPCs) [15]. Moreover, through a biomimetic progressive enhancement strategy, an anisotropic hydrogel with high strength, high toughness, excellent fatigue resistance, and good biocompatibility has been constructed, simulating the hierarchical structure of tendons [16]. A robust dual-layer Janus patch integrates an inner multifunctional electrospun hydrogel patch that provides antioxidant, anti-inflammatory, and antibacterial properties, with an outer poly-L-lactic acid layer serving as a physical barrier to prevent peritendinous adhesion [17]. Despite recent advancements, previous reviews have not comprehensively summarized the application of hydrogel formulations and their associated biological functionalities, such as responsiveness and targeting, in tendinopathy management.

This review aims to elucidate the use of hydrogels in tendinopathy from various perspectives, including hydrogel types, formulations, and biological functions, while integrating the pathophysiological mechanisms of tendon repair. It particularly emphasizes the preparation techniques of different hydrogel formulations, including material sources, crosslinking methods, responsive design considerations, and formulation strategies. Additionally, the review explores the repair mechanisms of hydrogels for injured tendons to provide a comprehensive reference for hydrogel applications in tendinopathy management.

## Pathophysiological Processes of Tendon Injury and Repair

### Overview of tendon structure and homeostasis

Tendons are primarily composed of highly aligned, collagen-rich proteins, which confer greater tensile strength compared to ordinary tissues [18]. Tendons connect muscles to bones and transmit the forces generated by muscle contractions to the skeleton system. Structurally, tendons exhibit a hierarchical organization ranging from the nanometer to the centimeter scale. Their extracellular matrix (ECM) is densely packed with a fibrous network. This network consists primarily of parallel-aligned type I collagen (Col I) fibers [19]. Tenocytes produce soluble procollagen molecules. These molecules are then crosslinked into insoluble collagen, which aggregates into microfibrils and finally assembles into microscopically visible collagen fibrils. These fibrils organize into bundles that serve as the fundamental structural units of tendons, known as collagen fibers. The collagen fibers integrate with the surrounding delicate connective tissue layer, the endotenon, to form primary fiber bundles (subfascicles), which aggregate into secondary fiber bundles (fascicles). These secondary bundles further assemble into tertiary bundles, forming the complete tendon architecture [20].

Although the human body contains over 600 muscle–tendon units, tendon disorders primarily affect 2 distinct anatomical regions: the upper limbs (particularly the shoulders and elbows) and the lower limbs (mainly the knees, ankles, and hips). Tendon injuries in the upper limb are frequently occupational in origin and commonly associated with repetitive movements of the upper extremity in specific job roles. In contrast, lower limb tendon injuries are predominantly observed in professional athletes and are attributed to high-demand loading conditions and repetitive tendon loading encountered during sports activities [21]. Tendons demonstrate a notably poor intrinsic healing capacity due to their hypovascular nature, with blood supply primarily restricted to the outer surface, where vascularization is most prominent. Simultaneously, the intratendinous circulation remains relatively sluggish. This insufficient blood perfusion has been recognized as a critical factor in delayed tendon healing. However, spontaneous healing of healthy tendon tissue can occur when the ruptured ends remain in contact and the well-vascularized paratenon (also referred to as peritendinous tissue) remains intact.

Tendon healing is a complex process that requires the coordinated action of cells, ECM, cytokines, and other proteins. Acute tendon injuries typically refer to tendon ruptures caused by acute trauma or spontaneous ruptures resulting from tendinopathy, which has been identified as a major contributor to tendon rupture [22]. Following injury, 2 distinct healing mechanisms are recognized: intrinsic healing refers to the activation of resident tenocytes, which proliferate and synthesize new collagen. Extrinsic healing involves the migration of inflammatory cells and fibroblasts from surrounding tissues like the sheath and synovium [23]. Tendon growth, development, and homeostasis are primarily regulated by 3 key transcription factors: scleraxis (SCX), mohawk homeobox (MKX), and early growth response 1 (EGR1). SCX, which is expressed in tendon progenitor cells, plays a central role in regulating early tendon growth and development. The transcription of Col Iα1, Col Iα2, aggrecan, and tenomodulin (TNMD) has been recognized to be dependent on SCX, while MKX is primarily involved in tendon maturation [24]. EGR1 facilitates the expression of tendon-specific marker genes, particularly SCX and Col Ia1, and actively contributes to tendon morphogenesis, homeostasis regulation, and repair processes [25].

### Phases of tendon healing

According to the classical perspective of tendon repair, the healing process is characterized by distinct, temporally dependent stages in which injured or damaged tendons progress through 3 primary recovery phases. Each phase involves specific cellular and molecular cascades regulated by distinct molecular and cellular compartments. These phases are mediated by different cell types, cytokines, and growth factors, and although they may overlap, their duration varies depending on the anatomical location and severity of the injury [26].

#### Inflammatory phase

Following tendon rupture, an acute inflammatory response persists for 3 to 7 days. During this period, pro-inflammatory cytokine gene expression at the injury site increases several thousand-fold within 2 to 3 days. This surge is accompanied by an infiltration of circulating inflammatory cells, such as macrophages, monocytes, and neutrophils. In this phase, blood vessels around the damaged tissue rupture. This causes blood to leak into adjacent areas, which triggers a further inflammatory response. This response is characterized by the accumulation of leukocytes, inflammatory cells, and growth factors [27,28]. Neutrophils and M1 macrophages are responsible for killing pathogens and performing cellular debridement of dead or dying cells, thereby facilitating the deposition of new ECM and the recruitment of cells. M2 macrophages contribute by phagocytosing tissue debris, depositing ECM, and secreting growth factors that activate stem and progenitor cells [29]. Among all inflammatory cell types involved in the inflammatory phase, macrophages are considered to play a key regulatory role in the healing process. Zhang et al. [30] have categorized macrophages into 3 subsets: MP1, MP2, and MP3. The MP1 subset comprises pro-inflammatory macrophages, characterized by elevated interleukin (IL)-1β and epiregulin expression, and plays an active role in mediating inflammatory responses. MP2 macrophages exhibit a pro-fibrotic phenotype, marked by high levels of secreted phosphoprotein 1 and matrix metallopeptidase-9 (MMP-9). In contrast, MP3 macrophages demonstrate anti-fibrotic characteristics, marked by the expression of folate receptor beta and lymphatic vessel endothelial hyaluronan receptor 1. Furthermore, these cells secrete cytokines that recruit resident cells near the injury site, subsequently initiating the synthesis of ECM components and tissue repair. Persistent inflammatory damage can impair healing and lead to chronic tendinopathy. However, a properly regulated early inflammatory response is indispensable for initiating tendon repair [12,31].

The initial phase of tendon healing is characterized by hematoma formation and the release of pro-inflammatory cytokines. These cytokines facilitate the recruitment of neutrophils, macrophages, and monocytes to the injury site. During this stage, infiltration of inflammatory cells occurs, initiating tenocyte migration, subsequent tenocyte proliferation, and type III collagen (Col III) production. The secreted angiogenic factors initiate the formation of a vascular network, which is essential for the viability of newly formed fibrous tissue [32]. Subsequently, ECM components synthesized by fibroblasts, primarily Col III, begin to repair the damaged tissue, signifying the commencement of tendon healing. Extensive infiltration of inflammatory cells during the early repair phase often results in adhesion formation, matrix degradation, and cell death, impeding effective tendon healing [33]. These adverse outcomes are attributed to increased matrix degradation and inflammation-associated factors by activated fibroblasts. Additionally, localized inflammation at the tendon defect site may induce elevated exudation, exacerbating fibrin leakage and promoting adhesion formation.

#### Proliferative phase

Following the inflammatory phase, tendon healing enters the proliferative stage. This stage is characterized by fibroblast proliferation, excessive ECM production, TSPC activation and tenogenic differentiation, and substantial vascular and neural ingrowth. Previous studies have reported that vascular endothelial growth factor (VEGF) and fibroblast growth factor beta (FGF-β), when administered either individually [34] or in combination [35], can enhance tendon healing. They act by promoting the proliferation of mesenchymal stem cells (MSCs), facilitating their differentiation into tenogenic lineages, increasing vascularization, and improving the mechanical strength of regenerated tendons. ECM synthesis is mainly conducted by fibroblasts recruited to the injury site, with Col III as the predominant component. The ECM produced during this phase is characterized by randomly organized proteoglycans and collagen fibers, particularly Col III, accompanied by elevated cellularity and substantial water retention. Fibroblasts continue to proliferate and migrate toward the injury site, while cells from the endotenon and epitenon also undergo active proliferation [36]. In addition, fibroblasts derived from the tendon sheath and synovium, along with tenocytes from the epitenon and endotenon, are recruited to the injury area and initiate proliferation. These cells contribute to excessive synthesis and random deposition of Col III and other ECM components, thereby facilitating the formation of an early provisional matrix. Concurrently, tendon-derived stem cells (TDSCs) are activated and contribute to the repair process. Recent studies employing single-cell transcriptomics and lineage tracing methodologies have identified Tppp3^+^Pdgfra^+^ cells as potential tendon stem cells (TSCs) capable of generating tenocytes and exhibiting self-renew capacity following injury [37]. During the proliferative phase, various growth factors, including basic FGF (bFGF), TGF-β, and insulin-like growth factor-1 (IGF-1), are continuously released at the injury site. These growth factors enhance cellular proliferation and angiogenesis, promoting adequate ECM production [38]. During this stage, neutrophil levels progressively decrease [39], while the initially predominant M1 macrophages gradually undergo polarization toward the M2 phenotype, which helps mitigate inflammation and reduce scar formation [40]. M2 macrophages continue to secrete growth factors that regulate cellular recruitment and function. The cytokine microenvironment at this phase stimulates tenocytes to produce Col III and other ECM components, thereby facilitating the formation of a new tissue matrix that supports the repair process. Simultaneously, angiogenesis, defined as the formation of new blood vessels, occurs to ensure adequate delivery of essential nutrients and oxygen to the healing tissue [39,41].

#### Remodeling phase

The remodeling phase comprises 2 distinct substages that commence approximately 6 to 8 weeks following injury and typically persist for 1 to 2 years, depending on the patient’s age and condition. The initial substage, known as consolidation, is characterized by a reduction in cellularity and matrix production as tenocytes and fibroblasts progressively decline in number and metabolic activity. This decline results in diminished synthesis of ECM components and cellular structures. Despite the overall reduction in ECM synthesis, the production of Col I increases and progressively replaces Col III. Tenocytes and collagen fibers, initially randomly arranged at the injury site, begin to realign along tension lines during the remodeling process. Besides, enhanced crosslinking within collagen fibers improves the tensile strength of regenerated tissue. However, the mechanical properties of repaired tendons rarely reach those of native tendon tissue due to the persistent presence of scar tissue. Two distinct modalities of tendon repair have been proposed: intrinsic healing, primarily mediated by tenocytes and epitenon-derived cells, is associated with scarless healing and superior functional recovery. In contrast, extrinsic healing, driven by fibroblasts and inflammatory cells migrating from peripheral tissues, results in scar formation and adhesion development. Optimal tendon repair requires a coordinated interplay between these 2 mechanisms throughout all 3 phases of healing. The initial phases are predominately governed by extrinsic healing, which facilitates the formation of fibrovascular scars. In contrast, intrinsic healing gradually becomes dominant, replacing scar tissue and promoting tendon regeneration with enhanced biomechanical properties and fewer complications. Nevertheless, the intrinsically low cellularity and vascularity of tendons restrict their inherent healing capacity. Consequently, excessive activation of extrinsic healing leads to various complications following tendon repair, including adhesions, rerupture, and repair failure [42,43].

Approximately 10 weeks following injury, the maturation phase initiates, characterized by enhanced crosslinking of collagen fiber and the formation of more mature tendon tissue. During the final stage, collagen fibers become more organized and aligned along the tendon’s fiber orientation. Consequently, the healing tissue progressively strengthens while the newly formed tissue matures and develops mechanical resilience. This process is driven by increased collagenase activity, which promotes the resorption of Col III and its replacement with Col I, accompanied by a reduction in cellularity within the repaired tissue. Accordingly, the resulting scar tissue collagen fibers exhibit improved organization and alignment, contributing to a stronger and more functional tendon. Concurrently, specialized cells such as macrophages actively degrade and remove excess scar tissue. This remodeling process may extend up to 1 year, during which the healed tissue progressively gains strength [44,45]. However, the metabolic activity of tenocytes and vascular cells progressively declines due to insufficient mechanical stimulation, scar tissue formation, and other contributing factors. This decline results in repaired tendons with inferior mechanical strength compared to healthy ones. Consequently, naturally healed tendons have poor functional recovery and a higher risk of rerupture [46]. Simultaneously, TGF-β signaling pathway is closely associated with tendon adhesion formation. This pathway can act synergistically with other pathways, including mitogen-activated protein kinases (MAPKs) and bone morphogenetic proteins (BMPs), to regulate essential physiological processes such as collagen synthesis and tissue remodeling. Within this pathway, Smad proteins 2 and 3 (SMAD2/3) perform dual functions: transcription factors for TGF-β and signal transducers within the TGF-β signaling cascade. The up-regulation of SMAD2/3 phosphorylation and sirtuin 1 promotes excessive TGF-β production in fibroblasts, enhancing ECM secretion and contributing to more pronounced tissue adhesions [47–49].

Heterotopic ossification represents a severe complication during tendon healing, distinct from scar formation, adhesion development, and healing failure. TSCs and MSCs can be driven to differentiate into chondrogenic and osteogenic lineages under mechanical stimulation, as indicated by elevated expression levels of chondrogenic and osteogenic markers. Dysregulation of critical microenvironmental factors may lead to loss-of-function or gain-of-function alterations in MSCs, impairing tissue regeneration or inducing ectopic bone formation [50]. Neutrophil overactivation causes immune dysregulation. This dysregulation triggers transforming growth factor beta 1 (TGF-β1) and BMP pathway activation in local tissues. Consequently, MSCs and progenitor cells differentiate into osteoblasts or osteoclasts, which facilitates heterotopic ossification [51]. Research has demonstrated that coculturing macrophages with osteoblast progenitor cells leads to enhanced activation of NF-κB signaling in macrophages. This activation promotes the secretion of BMP-6, BMP-2, and TGF-β1, which collectively activate osteogenesis and promote osteoblast differentiation [52]. During the inflammatory phase, recruited tendon-derived progenitor cells differentiate into chondrocytes and osteoblasts under various signaling pathways. For example, excessive activation of the hedgehog signaling pathway directs tendon-derived cells (TDCs) toward osteogenic differentiation, This shift is evidenced by the up-regulated expression of osteogenic genes, including alkaline phosphatase, bone sialoprotein, osteocalcin (Ocn), runt-related transcription factor 2 (Runx2), and Osterix [53]. Recent studies have identified clusters of differentiation 26 (CD26^+^) TSPCs as contributors to heterotopic ossification. In the tendon punch injury model, it has been demonstrated that during the late stage of tendon injury, CD26^+^ TSPCs can differentiate into osteoblasts and subsequently become embedded within the ectopic bone. Furthermore, the tenascin-C (TNC)-Hippo signaling pathway plays a critical regulatory role in mediating this process [54]. In a trauma-induced heterotopic ossification mouse model generated through percutaneous Achilles tendon puncture, Ocn^+^ osteoblasts were detected 6 weeks after injury, contributing to de novo bone formation, but they declined by 15 weeks. Furthermore, the number of tartrate-resistant acid phosphatase-positive (TRAP^+^) cells within the heterotopic bone increased 6 weeks following the puncture. Sustained osteoclastic bone resorption resulted in the formation of large marrow cavities by 15 weeks, at which point a few TRAP^+^ cells were observed [55].

Recent studies on neurovascular regeneration have revealed that following tendon injury, the expression of nerve growth factor (NGF) increases in macrophages and mesenchymal cells, reaching a peak at 7 days after injury. Anatomical regions exhibiting elevated NGF expression also display peripheral nerve innervation. TUBB3^+^ peripheral nerve fibers also demonstrate sprouting and disorganization surrounding the injury site. Alterations in the expression of the vascular marker CD31 follow a trend similar to that of nerve regeneration, and the rate of cell proliferation closely corresponds with neovascularization. During the proliferative phase, NGF binds to the tropomyosin receptor kinase A receptor on sensory neurons, activating downstream signaling pathways, including the TGF-β pathway, which promotes neurovascular responses and modulates tendon healing. In the remodeling phase, the growth of nerves and blood vessels largely returns to baseline levels. At this stage, newly formed nerves and blood vessels progressively stabilize, preserving normal function and structural integrity in the healed tendon [56].

## Classification of Hydrogel Formulation Approaches

Hydrogels are promising materials for tendon engineering, offering excellent biocompatibility and biodegradability, which enable them to create an ECM-like growth environment for cells [57]. By offering physical support and controlled drug delivery [58], hydrogels can play a crucial role in different stages of tendon healing [59]. Hydrogels can be classified into different types according to various categorization methods. Based on their material composition, they are divided into natural hydrogels, synthetic hydrogels, and semisynthetic hydrogels. Additionally, we have further classified hydrogels according to their size, morphological configuration, and crosslinking methods. Each category possesses unique characteristics tailored for specific biomedical applications. There exists a tightly coupled synergistic relationship among the raw materials, crosslinking methods, and morphological configuration/dimensions of hydrogels: the molecular structure of the raw materials directly determines the available crosslinking sites [e.g., sugar units containing abundant covalently bonded hydroxyl (-OH), carboxyl (-COOH), or amino (-NH₂), thereby providing extensive functionalization and subsequent modification possibilities] [60], which consequently influences the selection of crosslinking methods. Meanwhile, both the crosslinking density and uniformity collectively regulate the macroscopic morphological configuration and microscopic architecture of the hydrogels. The smaller the hydrogel mesh size, the lower the distance between adjacent crosslinks and the higher the crosslinking density. Additionally, a higher polymer volume fraction, smaller polymer molecular weight, higher crosslinker concentration, and longer crosslinking time all reduce the space between hydrogel copolymer chains available for water accommodation, resulting in a highly rigid structure. If the molecular weight of the polymer chains increases, the equilibrium swelling degree of the hydrogel also rises due to decreased crosslinking density and enlarged mesh size [61].

Physical crosslinking (hydrogen bonds/hydrophobic interactions) typically forms dynamic networks, while chemical crosslinking enhances mechanical strength. Dynamic covalent crosslinking (e.g., Schiff base bonds and disulfide bonds) further enables customization of complex shapes (biomimetic topological structures). Although Schiff base reactions represent a chemical crosslinking method, they still exhibit highly dynamic characteristics, making the networks more capable of recovery after deformation or damage compared to purely nondynamic chemical crosslinked networks [62]. Meanwhile, the hydrophilicity of raw materials can regulate the final dimensions through swelling behavior. For instance, in temperature-responsive hydrogels, hydrogen bonds are influenced by external temperature, leading to changes in both the hydrogel’s network structure and overall volume [63]. Moreover, the inherent biochemical properties of raw materials also critically determine the performance of resulting hydrogels. For instance, hyaluronic acid (HA) intrinsically possesses anti-inflammatory and pro-angiogenic characteristics [64]. In summary, hydrogels achieve performance matching through a closed-loop design paradigm of “raw materials–crosslinking methods–structure”, which establishes a theoretical foundation for the targeted development of smart hydrogels tailored to specific applications (e.g., stimulus-responsive crosslinked hydrogels for controlled drug delivery, or biomimetic topological structures for tissue engineering requirements).

### Material source of hydrogel formulations

Natural hydrogels have gained substantial attention in tissue engineering and regenerative medicine due to their outstanding biocompatibility and bioactivity. These materials can effectively regulate cellular behavior by mimicking the natural ECM microenvironment, obviously enhancing cell adhesion, proliferation, and tissue repair and regeneration. Their core components originate from natural biomacromolecules, primarily categorized into polysaccharides and proteins. Polysaccharides include HA, sodium alginate (SA), chitosan (CS), and agarose, which provide unique physicochemical properties. Proteins such as collagen, tropocollagen, gelatin, and silk fibroin (SF) contain biomimetic cell recognition sites [65]. However, traditional natural polymer hydrogels are inherently limited by weak mechanical properties and single functionality, severely restricting their application as structural tissue engineering scaffolds. Essentially, natural polymers are hydrophilic macromolecules, and due to the competition of hydrogen bonds between water and the polymer, the high-water affinity often disrupts intermolecular hydrogen bonds even when the network is chemically crosslinked [66]. Additionally, although natural hydrogels are safe for most people, in rare cases, certain materials in natural hydrogels can act as allergens [67].

Synthetic hydrogels are produced by crosslinking hydrophilic synthetic polymers, such as poly(ethylene glycol) (PEG), polyvinyl alcohol (PVA), polyacrylic acid and its derivatives, poly(lactic-co-glycolic acid) (PLGA), and poly(2-hydroxyethyl methacrylate). Compared with natural polymers, synthetic polymers have clear molecular weights and basic structural units, and their porosity, degradation time, tensile strength, and other properties can be precisely customized through predesign. Synthetic hydrogels offer reliable raw material sources, a longer shelf life, a broader variety of options, and stable performance. However, the synthesis process may introduce chemical crosslinkers and other toxic components. Additionally, synthetic hydrogels generally exhibit poorer degradability and biocompatibility [68].

Although natural polymers, such as various polysaccharides, have undoubted advantages related to their biocompatibility, biodegradability, and low cost, they are inferior to synthetic polymers in terms of water absorption and water retention properties. In this regard, the most promising are semisynthetic polymeric superabsorbents based on natural polymers modified with additives or grafted chains of synthetic polymers, which can combine the advantages of natural and synthetic polymeric hydrogels without their disadvantages [69].

#### Natural polymers

HA is a natural polymer that belongs to a group of heteropolysaccharides known as glycosaminoglycans (GAGs). It is found in various human tissues including the vitreous body, joints, umbilical cords, skin, and connective tissues [70]. HA consists of repeating disaccharide units of alternating β-1,4-D-glucuronic acid and β-1,3-N-acetyl-D-glucosamine. This unique structure endows it with high hydrophilicity and water retention capacity, enabling it to bind large amounts of water molecules and create a highly hydrated environment. As an essential component of the ECM, HA plays critical roles in various biological processes including cell signaling, morphogenesis, and matrix organization [71]. The synovial fluid within the tendon sheath serves as a vital exogenous nutritional source rich in HA, playing a pivotal role in maintaining overall tendon gliding function while simultaneously enhancing intrinsic tendon healing through nutrient provision. HA is predominantly synthesized by type B synoviocytes distributed throughout the synovial sheath [72]. Furthermore, owing to its negative charge, HA can effectively inhibit fibroblast growth, cellular proliferation, migration, and adhesion. Its unique 3D architecture enables controlled slow release of encapsulated substances through modulation of degradation rates for external hydrophobic groups. These distinctive properties allow HA to serve dual functions: as both a therapeutic dressing for injured tendons and an advanced drug delivery vehicle. This biomaterial achieves synchronized therapeutic effects by controlling drug release kinetics while physically preventing adhesion tissue formation at the structural level [73,74]. Hydrophobically modified nucleic acids (e.g., cholesterol-modified) can bind to hydrophobic pockets within hydrogels via hydrophobic interactions. For instance, cyclodextrin-modified HA hydrogels enable sustained release of cholesterol-modified miRNA over 3 weeks through such hydrophobic associations, maintaining in vivo efficacy for up to 14 days [75]. In summary, HA plays a pivotal role throughout the tendon tissue repair and healing cascade.

CS is a linear polysaccharide polymer derived from the N-deacetylation of chitin, exhibiting excellent biocompatibility and low cytotoxicity. It possesses inherent antimicrobial properties, demonstrating bactericidal effects against pathogens such as *Escherichia coli* and *Staphylococcus aureus*. Furthermore, the -NH₂ and -OH in its structure can react with free radicals, enabling radical-scavenging capabilities [76]. Moreover, due to its positively charged structure, CS can interact with anionic substances on red blood cell surfaces, inducing erythrocyte aggregation and fibrin clot formation [77]. CS can interact with various negatively charged biological macromolecules, including lipids, proteins, DNA, and cellular receptors, thereby inducing a cascade of interconnected biological responses in vivo that demonstrate its superior functional properties [78]. Adhesion to tissue was achieved by unilateral coupling of the amine rich bridging polymer CS to the dissipative alginate acrylamide hydrogel [79]. The -NH₂ in CS molecules undergo protonation and deprotonation under different pH conditions, resulting in a positively charged structure in acidic environments that causes hydrogel network expansion and absorption of negatively charged substances. In alkaline environments, deprotonation occurs, triggering the release of absorbed materials. This pH-responsive characteristic has been widely utilized in drug delivery systems. The porous structure provides storage space for drugs, while the stimulus responsiveness enables precise control over drug release [80]. CS-crosslinked mussel-inspired materials can enhance the mechanical strength of artificial tendons derived from mussel proteins while providing controllable degradation [81]. This approach not only accelerates functional tendon reconstruction but also enables harmless clearance post-implantation.

Alginate is a linear anionic polysaccharide derived from brown algae or bacteria, exhibiting excellent biocompatibility with minimal immunogenicity. It provides an optimal microenvironment that facilitates cell adhesion, proliferation, and differentiation, thereby promoting tissue repair. Notably, alginate demonstrates remarkable water-absorbing capacity, enabling effective absorption of both moisture and exudate. This characteristic helps prevents desiccation and accelerates tissue healing while simultaneously removing excess exudate to reduce infection risks [82]. Additionally, alginate’s excellent histocompatibility makes it suitable for encapsulating cells and organelles. The resulting hydrogel possesses a porous structure that provides an optimal scaffold for cell adhesion, proliferation, and migration. This architecture facilitates efficient exchange of nutrients and gases, thereby creating favorable conditions for tissue regeneration [83].

Collagen exhibits excellent cytocompatibility, biological adaptability, nontoxicity, and biodegradability. Its viscoelastic properties allow fiber sliding through the disruption of weak intermolecular interactions under stress. Notably, collagen hydrogels with varying viscoelasticities influence cell adhesion, morphology, and actin organization. At physiological temperatures, collagen demonstrates self-assembling capability to form fibrous hydrogels. These 3D culture matrices maintain outstanding biocompatibility with cells, making them particularly suitable for encapsulating MSCs during in vitro cultivation [84]. Recombinant Col III, an essential component of the ECM, is produced through optimized expression of human collagen genes. This bioengineered protein demonstrates high biocompatibility and low immunogenicity, creating an ideal microenvironment for cell growth and tissue repair. It actively promotes fibroblast and endothelial cell migration, and enhances angiogenesis [85]. This biomaterial demonstrates potential for mimicking the tendon ECM to promote tendon cell adhesion and aligned organization while improving vascular supply. The CS–collagen composite effectively delays collagen degradation rates and enhances scaffold mechanical strength [86], thereby maintaining sufficient therapeutic duration and providing optimal mechanical support.

Gelatin is a protein-based biomaterial derived from partial hydrolysis or thermal denaturation of collagen, primarily sourced from porcine, bovine, piscine, and poultry tissues including bones, skin, tendons, ligaments, and connective tissues. Characterized by its inherent Arg-Gly-Asp sequences, this material exhibits excellent cell adhesion properties that promote cellular attachment, proliferation, and differentiation while demonstrating reduced antigenicity. Furthermore, gelatin displays stimuli-responsive behavior with sensitivity to environmental changes such as temperature and pH variations [87]. The formation of a double-network structure, consisting of a chemically crosslinked polyacrylamide (PAM) network and a physically crosslinked gelatin network, enhances mechanical strength [88], providing a valuable reference for adapting to high-intensity tendon stresses and promoting tendon regeneration. Interestingly, gelatin serves as an attractive substrate for designing “smart” hydrogels for drug delivery [89], enabling localized administration and reduced drug side effects.

SF exhibits excellent biocompatibility and provides an optimal microenvironment for cell survival, facilitating cell adhesion, proliferation, and differentiation. Due to its unique hierarchical structure primarily determined by β-sheet microcrystals, it demonstrates superior mechanical properties compared to other biomaterials. It exhibits exceptional fracture strain, strength, and toughness that surpass many synthetic fibers [90]. Its mechanical properties vary with concentration-in composite hydrogels prepared with gelatin, and the compressive modulus increases proportionally with SF content. This tunable mechanical behavior enables the hydrogel to better accommodate diverse tissue regeneration requirements [91]. SF hydrogels demonstrate excellent degradability both in vitro and in vivo, and are considered fully bioabsorbable. This property eliminates the need for secondary surgeries after tendon tissue implantation, thereby reducing patient discomfort.

#### Synthetic polymers

Polydopamine (PDA) exhibits high biocompatibility, low cytotoxicity, and biodegradability, along with metal ion chelation capabilities that enable binding with metallic species. The active catechol groups on PDA can serve as building blocks for biomaterials through metal chelation effects, hydrogen bonding, and electrostatic interactions, providing effective modification sites. Metal ions and antibacterial agents can be conjugated with PDA to construct antimicrobial interfaces. For instance, copper ions (Cu^2+^) binding with PDA enables controlled release of Cu^2+^, achieving mild yet persistent antibacterial efficacy [92]. This approach may offer novel strategies for preventing postoperative tendon infections. Additionally, PDA demonstrates efficient photothermal conversion, transforming light energy into heat, which enables photothermal therapy applications. In sports injury treatment, this localized heating effect can enhance blood circulation while alleviating muscle spasms and pain [93]. PDA can polymerize and deposit under mild conditions, can bind to molecules containing free -NH₂, and exhibits good stability [94]. PDA possesses abundant amine, imine, and aromatic ring groups that enable efficient drug loading and targeted delivery through chemical reactions or π–π stacking interactions. These active functional groups can interact with drug molecules to achieve stable drug loading on PDA-modified carriers, thereby enhancing drug bioavailability and therapeutic efficacy. Additionally, PDA’s aromatic rings and hydrophilic catechol groups exhibit antioxidant properties capable of scavenging free radicals and mitigating oxidative stress-induced inflammatory responses. In addition, its unique photothermal effect can suppress inflammatory cell activity and reduce pro-inflammatory cytokine release, while also promoting macrophage M2 polarization to improve the local immune microenvironment for tissue repair [95]. Building upon these properties, PDA may demonstrate potential in enhancing tendon vascularization, alleviating oxidative stress during the inflammatory phase, and accelerating the transition from the inflammatory to remodeling phase in tendon healing.

PEG exhibits excellent hydration capacity, environmental inertness, minimal protein adsorption, limited cell activation/adhesion, nontoxicity, and strong biocompatibility. Its outstanding biocompatibility and high aqueous solubility make it biologically inert with low protein absorption, rendering it an ideal scaffold for nanoparticle and drug loading [96]. Moreover, it demonstrates rapid gelation, strong tissue adhesion, on-demand dissolubility, and excellent biocompatibility [97]. It shows promise as a raw material for developing patch hydrogels with drug delivery capabilities, potentially facilitating tendon repair. Furthermore, it can be formulated into injectable hydrogels that enhance cell migration rates. The 3D porous network structure and high specific surface area of these hydrogels create a more favorable microenvironment for cell proliferation and migration, while facilitating nutrient transport, metabolic waste removal, and tissue regeneration [98].

PAM has become invaluable in the design of hydrogel scaffolds for tissue engineering due to its unique mechanical and biological properties, as well as its ability to exhibit collective behaviors. The material exhibits water retention, electrical conductivity, stability, stretchability, and actuation capabilities [99]. Due to its exceptional hydrophilicity and nontoxicity, PAM has become a widely utilized synthetic polymer in hydrogel synthesis [100], demonstrating broad application prospects in biomimetic tissue engineering.

PVA is a water-soluble polymer synthesized through vinyl acetate polymerization and subsequent alcoholysis. Its abundant hydrophilic groups confer exceptional moisture absorption capacity, while enabling complete biodegradation into water and carbon dioxide. PVA has become a very promising biomedical material due to its excellent properties such as biodegradability, outstanding biocompatibility and nontoxicity [101]. As a scaffold material for tissue engineering, PVA provides structural support for cell growth and differentiation while promoting tissue repair and regeneration. The material also serves as an effective drug delivery vehicle, enabling controlled sustained release of therapeutic agents to enhance treatment efficacy while reducing systemic side effects [102]. PVA hydrogels can be engineered to mimic the mechanical strength of human soft tissues due to their exceptional stability across a wide range of temperatures and pH conditions. Beyond biocompatibility, PVA exhibits a unique combination of properties: water solubility, film-forming capability, adhesiveness, and chemical resistance. However, PVA exhibits certain limitations in biomedical applications, such as brittleness and thermal degradation. Nevertheless, the brittleness of PVA can be mitigated by adding plasticizers like PEG, which reduces its stiffness and enhances flexibility. Techniques such as high-density hydrogen bonding, chain entanglement networks, and the salting-out effect via sodium chloride immersion can be employed to modify PVA hydrogels, further improving their mechanical properties. These methods enhance the tensile strength, elastic modulus, and toughness of PVA [103], matching the mechanical environment required by tendons as high-tension, load-bearing tissues.

Carboxymethyl cellulose (CMC) exhibits unique swelling behavior and tunable mechanical properties. CMC can be prepared from bacterial cellulose, which has high purity and does not require chemical treatment to remove impurities such as hemicellulose and lignin, making it an environmentally sustainable source. It possesses a unique 3D network structure and nanoscale fibers, providing an excellent foundation for preparing high-performance hydrogels. Upon swelling, it forms stable transparent gels capable of holding large amounts of water. Due to its high purity, stability, and water absorption capacity [104], it shows promise as a dressing to provide a moist tissue-healing environment, deliver controllable mechanical support, or serve as a carrier material for controlled drug delivery.

#### Semisynthetic polymers

Acetylated HA (AcHyA) combines the bioactivity of HA with the stability of synthetic polymers. HA has inherent limitations in scaffold construction due to its physicochemical properties, including difficulties in electrospinning and low cell adhesion rates. Acetylation modification reduces HA’s high negative charge density and strong hydrophilicity, enabling solubility in highly polar organic solvents. This addresses HA’s challenges in electrospinning processes and allows for the preparation of nanofiber composite scaffolds when blended with polycaprolactone (PCL), expanding its applications in tissue engineering [105]. Furthermore, the introduction of acetyl groups enhances the lipophilicity of the polymer backbone, thereby improving its absorption and retention in vivo. This acetylation modification increases the bioavailability of HA. Both AcHyA and native HA exhibit excellent antioxidant and anti-inflammatory properties, with AcHyA demonstrating superior performance compared to native HA. While maintaining HA receptor binding capacity, the increased hydrophobicity of AcHyA may influence cell–microenvironment interactions, subsequently modulating cellular functions [106].

Gelatin methacryloyl (GelMA) is synthesized by the direct reaction of gelatin with methacrylic anhydride in phosphate buffer at 50 °C, which combines the bioactivity of gelatin with the mechanical properties of synthetic polymers. The physical characteristics of GelMA can be precisely controlled by adjusting both the degree of methacrylation and gel concentration. This material exhibits excellent pattern fidelity and cellular responsiveness, making it highly suitable for various microscale tissue engineering applications, including the fabrication of complex microtissues such as endothelialized microvascular systems [107]. GelMA is modified from gelatin, which is a derivative of natural collagen. Gelatin possesses advantages such as no adverse reactions, no immunogenicity, and good biodegradability, giving GelMA excellent biocompatibility that provides a suitable growth environment for cells. This makes it applicable as a drug carrier or for implantation in organisms. By adjusting the degree of methacrylation and gel concentration, the mechanical properties of GelMA hydrogels can be regulated. Increasing the degree of methacrylation or gel concentration enhances the stiffness and compressive modulus of the hydrogel, allowing it to meet the mechanical requirements of different tissue engineering applications [108]. Furthermore, cells can be suspended in the GelMA prepolymer solution and photo-crosslinked upon ultraviolet (UV) exposure to form 3D cell-encapsulating hydrogels. Unlike 2-dimensional (2D) cell culture systems, hydrogel-embedded cells demonstrate microenvironment-remodeling capabilities to enable cellular spreading and migration [109]. In summary, the mechanical properties of GelMA can potentially mimic the viscoelasticity of natural tendons, playing a role in tendon engineering by delivering drugs or cells to promote tendon repair and regeneration.

### Size and morphological configuration of hydrogel formulations

Hydrogels can be categorized by size and morphological configuration into macroscopic hydrogels and hydrogel microspheres (MSs). Based on morphological configuration variations, macrogels are further classified into cylindrical, porous sponge-like, fibrous, membrane-like, spherical forms, among others. Higashi et al. [110] designed a cylindrical hydrogel based on cyclodextrin and PEG (potentials of polypseudorotaxane hydrogels) for the stabilization and delivery of high-concentration human immunoglobulin G (IgG). This system can form stable hydrogels in the presence of high IgG concentrations. The cylindrical structure enables physical crosslinking, providing a stable sustained-release system. Porous sponge-like hydrogels exhibit a surface morphology characterized by interconnected porous structures, where the increased specific surface area enhances drug loading capacity and facilitates sustained release [111]. Fibrous hydrogels, fabricated via electrospinning technology, possess a fiber-like structure resembling the natural ECM. This architecture provides an optimal environment for cell attachment and growth, facilitating tissue repair and regeneration. Additionally, these hydrogels exhibit excellent biocompatibility and biodegradability, allowing gradual absorption by the body after fulfilling their reinforcement functions, thereby avoiding potential complications associated with long-term implantation [112]. The self-assembling nanofiber hydrogels encapsulate cells in a manner analogous to natural 3D ECM, demonstrating reparative effects on senescent or degenerated human TSPCs [113]. Membrane-like hydrogels exhibit stable drug release characteristics, achieving triphasic sustained release of IL-4 through surface release, channel release, and MSs degradation release. These hydrogels demonstrate high water absorption capacity, effectively absorbing excess exudate while maintaining excellent conformability. Furthermore, they promote macrophage polarization toward the anti-inflammatory phenotype, suppressing secretion of pro-inflammatory factors such as tumor necrosis factor-α (TNF-α) while up-regulating expression of anti-inflammatory factors (e.g., TGF-β and PDGF) [114]. The results demonstrate that hydrogels with distinct geometries exhibit respective advantages—each topology optimally promotes cellular proliferation, enhances cell adhesion, and achieves sustained drug release, collectively offering promising applications for tendon repair.

Microgels encompass micrometer-scale MSs and nanometer-scale MSs. Particulate hydrogels with particle sizes in the micrometer and nanometer ranges are termed microgels and nanogels, respectively. Microgels and nanogels can be directly injected as they are smaller than the inner diameter of syringe needles compared to macroscopic hydrogels. Additionally, their larger relative surface area enhances the ability to penetrate tissue barriers and facilitates natural clearance. Zuo et al. [115] developed highly permeable micro/nano hydrogel MSs with an average particle size of 185 μm. These MSs can be uniformly dispersed in ultrapure water and administered via syringe injection, meeting the requirements for intra-articular delivery. Importantly, they demonstrate good retention stability in the articular cavity microenvironment. Their cationic surface charge enables effective penetration through the cartilage matrix via electrostatic interactions, ultimately reaching the subchondral bone. Moreover, the nanogels exhibit a distinct internal porous structure that forms a 3D network configuration. The raw materials show excellent compatibility without noticeable delamination, which facilitates cell adhesion, proliferation, and efficient exchange of nutrients and metabolic by-products. Simultaneously, these microgels enable sustained oxygen release to maintain intracellular oxygen levels, thereby promoting cell survival and growth [116]. These hydrogels show promising potential for addressing vascular cell deficiency following tendon injuries, offering a viable therapeutic strategy.

### Crosslinking methods of hydrogel formulations

#### Physical crosslinking

Physical crosslinking normally leads to a rapid polymerization behavior under relatively mild conditions and needs no toxic crosslinkers or catalysts, decreasing potential cytotoxicity. Since physical interactions are reversible, they have the potential to design injectable hydrogels with the capacities of shear-thinning and self-healing for extrusion bioprinting and injection [117]. Physical crosslinking does not require additional crosslinking agents and can spontaneously form gels by altering external conditions of the precursor solution. Hydrogels formed through this approach are typically nontoxic or have low toxicity. Furthermore, physically crosslinked hydrogels often exhibit self-healing properties, injectability, and stimulus responsiveness [118]. However, their relatively low mechanical properties and poor stability limit their clinical applications.

To enhance their mechanical performance, combinations of different crosslinking methods are typically employed. Ionic interactions involve the electrostatic attraction between polyelectrolytes and oppositely charged substances. However, these hydrogels exhibit relatively weak mechanical properties and limited stability in physiological environments. Additionally, the release of ions during the degradation of ionically crosslinked hydrogels may induce biotoxicity. The selection of ion types and concentrations is therefore critical. Hydrogen bonds refer to interactions between hydrogen atoms and electronegative atoms (such as nitrogen, oxygen, or fluorine), involving dipole–dipole bonding. Due to their unique directionality, tunability, and specificity, hydrogen bonds are widely utilized for crosslinking hydrogels. However, hydrogels formed solely through hydrogen bonding typically exhibit poor mechanical strength and are prone to fracture. To enhance mechanical strength, multiple hydrogen bonds can be introduced or other crosslinking methods may be incorporated [119].

Self-assembly refers to the process where components spontaneously organize into specific patterns or structures without external intervention, a mechanism that has become a powerful approach for constructing diverse functional materials. The spontaneous and noncovalent nature of this self-assembly process typically endows the interactions with reversible characteristics. Self-assembly serves as an effective strategy for fabricating hydrogels, where small molecules can form physically crosslinked hydrogel networks through noncovalent self-organization. These materials are formed through reversible dynamic bonds between specific components, enabling them to dissipate mechanical stress via structural reorganization and achieve self-healing after destructive mechanical impacts [120].

#### Chemical crosslinking

Compared with physically crosslinked hydrogels, chemically crosslinked hydrogels usually have covalent bonds among polymer chains, and most of their linkages are generally more substantial and more permanent than their counterparts. So far, these chemically crosslinked hydrogels have resulted from polymerization-induced cross-linking, enzyme-induced cross-linking, Diels–Alder “click” reactions, Schiff bases, etc. Further, chemically crosslinked hydrogels, on the other hand, are generally more stable under physiological conditions and exhibit exceptional mechanical characteristics and tunable degradation behavior [121].

#### Dynamic crosslinking

Dynamic crosslinking combines the characteristics of physical and chemical crosslinking, enabling hydrogels to undergo fracture and reversible reorganization under various stimuli. This unique property allows hydrogel materials to exhibit self-healing capabilities, shape recovery, and intelligent responses to wound environments. Dynamic crosslinked hydrogels achieve network reconstruction through physical noncovalent interactions, including hydrophobic interactions, host–guest interactions, hydrogen bonding, etc. Additionally, dynamic crosslinked networks can utilize dynamic covalent bonds such as borate esters, imines, disulfides, free radical polymerization, acylhydrazones, and amide bonds to enhance the mechanical properties and bonding strength of hydrogels [122,123]. The tunability and reversibility of dynamic crosslinked hydrogels make them a versatile, customizable, and sustainable material platform. In tissue engineering, they are used to construct artificial tissues and organ scaffolds, promoting cell proliferation and differentiation. Furthermore, dynamic crosslinked hydrogels can serve as drug delivery systems. By adjusting the dynamic properties and drug-loading capacity of hydrogels, controlled and targeted drug release can be achieved, improving therapeutic efficacy and safety. Moreover, they can be employed as biomimetic materials [124]. This dynamic crosslinking plays a pivotal role in the design of responsive hydrogels.

## Responsive Design Considerations of Hydrogel Platforms

Stimuli-responsive hydrogels are water-swollen polymer networks capable of undergoing volume phase transitions in response to external stimuli. They can react to various external triggers by altering their structure, physical composition, chemical properties, or mechanical performance. Based on the type of stimulus, hydrogels are primarily categorized into 3 classes: physically responsive hydrogels, chemically responsive hydrogels, and biochemically responsive hydrogels. Through the strategic design of polymer molecules, the properties of hydrogels can be modified, making them increasingly “intelligent” [125,126].

Cells can sense and respond to their surrounding matrix, while in turn, the physicochemical properties of the ECM can continuously influence cellular biological events. The physical properties of hydrogels, including stiffness, pore size, viscoelasticity, structure, and degradability, can modulate cell biology by altering mechanical transduction signals. Furthermore, the chemical properties of hydrogels, such as cell attachment sites, chirality, hypoxia-inducible functional groups, and reversible crosslinking sites, can regulate integrin clustering and subsequent signal cascades, thereby ultimately determining cell fate. Stimuli-responsive hydrogel systems designed to control cellular biological behavior can specifically react to external stimuli like light, electricity, pH, temperature, and ionic strength. These systems employ spatiotemporally precise reversible/irreversible regulation mechanisms to achieve on-demand modulation of biochemical and mechanical signals, enabling real-time manipulation of 2D or 3D cellular microenvironments to mediate cell behavior and function. Moreover, such stimuli-responsive hydrogels have found broad applications in targeted drug delivery systems [127].

Responsive hydrogels combine the biocompatibility and stretchability of natural hydrogels while overcoming the limitations of conventional materials. These hydrogels can precisely mimic native tissues and provide tunable mechanical properties, offering robust mechanical support for tissue repair. Moreover, they can effectively modulate endogenous bioelectrical activity to improve inflammatory microenvironments. At tendon injury sites, these smart hydrogels can actively sense local bacteria, fully exerting their antibacterial and infection-prevention potential. They enable sustained drug release and localized targeted delivery, enhancing drug utilization efficiency, prolonging therapeutic duration, and reducing adverse effects. In the field of tendon regeneration engineering, responsive hydrogels demonstrate highly valuable potential applications, and their roles should not be underestimated.

### Temperature-responsive hydrogels

Temperature-responsive hydrogels are soft materials formed through noncovalent physical crosslinking, whose crosslinked network formation and dissociation are regulated by temperature variations [128]. Thermosensitive polymers can undergo coil-to-globule conformational transitions in aqueous solutions [e.g., poly(N-isopropylacrylamide)] or demonstrate similar phase transition behaviors in organic solvents [e.g., polystyrene in cyclohexane and poly(methyl methacrylate) in acetonitrile], with their solutions exhibiting distinct temperature-responsive characteristics depending on chemical structures. By grafting these thermosensitive polymers with other biocompatible natural or synthetic polymers, the resulting phase transitions at body temperature enable both enhanced hydrophobic drug loading capacity through hydrophobic domains and effective encapsulation of bioactive molecules (e.g., growth factors and genes) via electrostatic interactions within hydrophilic grafted segments [129]. The selection of cell carriers constitutes a critical determinant for the success of tissue engineering-based approaches in tendon repair, where temperature-responsive hydrogels provide a 3D microenvironment supporting in vitro cell proliferation and matrix remodeling while serving as implantable cell delivery vehicles for in vivo applications. Moreover, these hydrogels can be injected in liquid form to completely fill target tissue defects with minimal invasiveness before undergoing thermally triggered gelation at physiological body temperature [130].

Temperature-responsive hydrogels achieve therapeutic effects by releasing encapsulated drugs or bioactive molecules in response to the temperature of injured tendons, while their gel state can simultaneously create a physical barrier to block the diffusion of inflammatory factors and prevent tendon adhesion formation, with these intelligent stimuli-responsive systems having been extensively synthesized and demonstrating application value in the field.

### pH-responsive hydrogels

According to different groups, pH-responsive hydrogels can be divided into anionic and cationic hydrogels and covalently bonded pH-responsive hydrogels, among which anionic and cationic pH-intelligent responsive hydrogels can be further divided into anionic and cationic pH-responsive hydrogels according to the charge properties of charged groups. Anionic pH hydrogels carry common negative charge groups [131]. By leveraging pH sensitivity, hydrogels can dynamically adjust drug release according to the local acidic or alkaline tissue environment, achieving precise treatment while minimizing drug release in noninflammatory areas, which improves therapeutic outcomes and reduces side effects. Additionally, pH influences the self-healing properties of hydrogels—under acidic conditions, the dissociation of Schiff base structures allows the hydrogel network to reform dynamic bonds, facilitating self-repair. pH responsiveness may also alter the mechanical properties of hydrogels [132]. The pH-responsive mechanism can also depend on borate ester bonds formed via reversible interactions between boric acid and diols, which are formed through reversible interactions between boric acid and diols. These bonds break under acidic conditions and reform in alkaline environments. In acidic settings, the protonation of amine groups (forming -NH₃^+^) causes the hydrogel network to swell. This protonation enhances the hydrogel’s hydrophilicity, further facilitating drug release [133]. pH-responsive hydrogels possess the unique capability to deliver materials or drugs to targeted sites where they can either form hydrogels or release their payload in situ in response to local tissue pH variations [134], making them particularly valuable for applications in drug delivery systems and as functional scaffolds for tendon tissue engineering. For instance, researchers have employed calcium ions (Ca^2+^)-crosslinked alginate hydrogels to deliver IGF-1 for tendon regeneration, observing that hydrogel swelling under low pH conditions facilitates IGF-1 release [135].

### Redox-responsive hydrogels

Redox-responsive hydrogels constitute a category of intelligent materials capable of modulating their physicochemical properties or structural configurations through dynamic responses to oxidation–reduction state alterations in their constituent molecular components. These hydrogels not only possess an aqueous matrix permitting the diffusion of water-soluble molecules, but also incorporate redox-sensitive chemical bonds (e.g., disulfide bonds and diselenide bonds) within their structure that respond to changes in the redox environment [136]. Disulfide bonds can cleave into thiol groups under reductive conditions, leading to disintegration and degradation of the hydrogel crosslinked network, with this redox-responsive characteristic enabling targeted drug release that enhances healing efficiency while minimizing off-target drug distribution. Moreover, the self-healing capability of such hydrogels partially originates from the presence of disulfide bonds, allowing network fracture under high strain but structural recovery at low strain to exhibit dynamic self-repair properties [137].

In tendons, which are hypovascular, hypocellular, and composed predominantly of ECM, reactive oxygen species (ROS) likely play a dual role: regulating cellular processes such as inflammation, proliferation, and ECM remodeling under physiological conditions, while contributing to tendinopathy and impaired healing when dysregulated [138]. Excessive ROS not only drives M1 macrophage polarization during the initial tendon repair phase, amplifying inflammatory responses and aggravating immune microenvironment imbalance to establish a vicious cycle of impaired tendon healing, but also demonstrates clinical relevance through superoxide-induced oxidative stress being directly associated with recurrent tear incidence following arthroscopic rotator cuff repair procedures [139]. Moreover, hydrogels can further encapsulate biomacromolecules and regulate protein release kinetics by adjusting the molecular weight of polymer precursors [140], enabling targeted sustained drug delivery in progressively damaged areas through coordinated control of precursor molecular weight and redox responsiveness to minimize side effects while enhancing therapeutic efficacy.

### Magnetic-responsive hydrogels

Magnetically responsive hydrogel systems are typically constructed by integrating polymeric matrices with magnetic functional units (such as superparamagnetic Fe_3_O_4_ nanoparticles), offering the unique advantage of precise noncontact regulation of physical properties, biochemical microenvironments, and mechanical characteristics through magnetic field stimulation. The smart responsiveness of these materials is primarily governed by 4 key parameters of the magnetic particles: composition type, concentration gradient, particle size distribution, and dispersion uniformity. Under an applied magnetic field, the magnetic particles form chain-like aligned structures due to magnetically induced aggregation effects, triggering a magnetically induced phase transition in the hydrogel network. The conformational changes in polymer chains lead to network contraction while solvent molecules undergo directional migration through a diffusion-percolation mechanism, ultimately achieving millisecond-scale dynamic deformation responses. This magnetically controlled dynamic behavior provides a new paradigm for precision medicine, enabling spatiotemporal regulation of drug release kinetics through programmed magnetic field parameters or in situ intervention in the proliferation and differentiation behaviors of loaded cells [126].

Beyond serving as physical scaffolds for cells, these biomaterials can also deliver essential mechanical stimulation through structural deformation induced by external magnetic fields, thereby creating dynamic microenvironments capable of triggering diverse cellular responses, while electromagnetic fields operate at the cellular level through multiple pathways to influence intercellular communication, regulate cytoskeletal organization, alter plasma membrane composition, and modulate intracellular calcium homeostasis [141]. Following tendon injury, the microscopic architecture demonstrates characteristic alterations including collagen fiber thinning and disorganization, accompanied by neovascularization within the tendon matrix and morphological transformations of tenocytes [142]. Consequently, the realignment of collagen fibers and restoration of tenocyte morphology represent hallmark features of successful tendon healing, which magnetic-responsive hydrogels may potentially enhance by promoting cellular alignment and differentiation, while their inherent safety profile and noninvasive nature position them as a promising therapeutic priority for tendon repair.

### Bioelectrically responsive hydrogels

Transcutaneous electrolysis can induce controlled inflammatory responses in tendons through nonthermal electrochemical reactions, potentially promoting biological processes that facilitate the regeneration of injured and healing tendons, with its application in Achilles tendinopathy specifically up-regulating the expression of genes associated with collagen regeneration and ECM remodeling, particularly cyclooxygenase 2 (COX-2), MMP-9, and VEGF [143]. Conductive hydrogels retain the inherent properties of conventional hydrogels while acquiring electrical conductivity through the incorporation of conductive materials, with conductive polymers demonstrating superior application advantages over traditional conductive materials due to their exceptional electrical performance, flexible mechanical characteristics, and unique capability to facilitate both electron conduction and ion transport in aqueous media [144]. Conductive hydrogels can facilitate organized cell alignment and modulate endogenous bioelectricity through their electrical conductivity, thereby improving inflammatory microenvironments and enhancing intracellular Ca^2+^ influx. These properties are of great important for promoting tissue regeneration [145]. Stretchable conductive polymer hydrogels exhibit excellent biocompatibility and stretchability without inducing inflammatory responses or fibrosis, making them suitable for long-term implantation in vivo. These materials can effectively replace damaged peripheral nerves, restore the conduction of bioelectrical signals, and reduce electrical resistance through photothermal effects, thereby enhancing bioelectrical signal transmission to promote nerve regeneration and functional recovery. Their combined photothermal responsiveness and electrical conductivity further facilitate the growth and differentiation of nerve cells, accelerating the neural regeneration process [146]. All these superior properties make them promising therapeutic solutions for tendon regeneration and repair.

### Enzyme-responsive hydrogels

Enzymatic substances such as MMPs and deoxyribonuclease, serving as crucial mediators for diverse biochemical reactions in organisms, provide natural triggering mechanisms for developing enzyme-responsive hydrogels through their specific catalytic functions, where enzyme–substrate integration approaches (as crosslinkers or functional side groups) differentially induce either bulk property modifications (e.g., crosslinking density variations) or localized group transformations (e.g., functional site activation) through enzymatic reactions. In both pathological and healing tendons, MMP expression exhibits dysregulation. Incorporating specific peptide domains into PEG hydrogels can transform the inherently inert synthetic PEG hydrogels into dynamically responsive MMP-sensitive hydrogels, while these peptide motifs can also be utilized to functionalize natural polymers like HA for developing MMP-responsive hydrogel systems beyond synthetic platforms [147]. The hydrogel network contains enzyme substrates that, upon binding with specific enzymes, can trigger degradation or property changes of the hydrogel. Composed of biocompatible materials, these hydrogels ensure safety for in vivo applications. By designing specific enzyme-responsive mechanisms, hydrogels can achieve targeted drug release in particular tissues or cells, enhancing therapeutic efficacy while minimizing side effects. For instance, glucose oxidase immobilized within hydrogels converts glucose into gluconic acid, lowering the local pH and inducing hydrogel swelling or degradation to release insulin [148]. MMPs, collagen-degrading enzymes synthesized and secreted by fibroblasts, are capable of cleaving structural molecules like collagen, playing indispensable roles in maintaining ECM homeostasis, while dysregulated MMP activity due to tendon injury or pathology disrupts normal ECM architecture, consequently impairing force transmission and leading to functional deficits [149]. Responsive controlled-release drug-loaded hydrogels regulate their degradation and drug release kinetics, paving the way for targeted tendon tissue repair.

### Microbial-responsive hydrogels

In rotator cuff tear repair surgery, postoperative infection is one of the critical factors leading to surgical failure and poor patient recovery. Skin secretion of *Andrias davidianus* (SSAD)-derived hydrogel demonstrates broad-spectrum antibacterial activity against both gram-positive and gram-negative bacteria by suppressing pathogen proliferation in vitro and controlling infection spread in vivo, thereby creating favorable infection control conditions for tendon repair [150]. The antibacterial mechanism of hydrogels relies not only on the inherent antimicrobial activity of their components but also on their responsive interaction with bacterial infections in the tissue microenvironment. These hydrogels can detect the presence of bacteria and dynamically modulate their properties to enhance antibacterial efficacy [151].

## Formulation Design of Hydrogel Platforms

To address the physical and biochemical needs of tendinopathy treatment, hydrogels are engineered in various formulations, including scaffolds, patches, sprays, MSs, and injectable systems. These platforms function as carriers for the delivery of drugs, cells, or bioactive compounds and serve as physical barriers and sources of mechanical support, among other functions.

For instance, the dual-crosslinked bioactive hydrogel patch possesses porosity, biodegradability, adjustable mechanical properties, and an intrinsic dual regulatory capability for the inflammatory microenvironment without the need for additional therapeutic agents [152]. Due to their excellent biocompatibility and degradability, porous GelMA hydrogel microspheres (GMs) serve as cell delivery carriers to facilitate the adhesion, proliferation, and infiltration of exogenous MSCs [153]. The injectable hydrogel formulation, capable of in situ formation through spontaneous intermolecular interactions, utilizes a phospholipid polymer-based hydrogel composition that can effectively isolate tendons from adjacent tissues and reduce the occurrence of adhesions [154].

### Hydrogel scaffolds

Gene-activated scaffold materials enable the localized secretion of growth factors or proteins to promote tissue regeneration. For example, 3D-printed scaffolds functionalized with plasmid DNA encoding therapeutic genes achieve genetic activation and facilitate tissue repair at the defect site. Similarly, gene-activated CS/Col scaffolds can stimulate cellular growth and differentiation, enabling in situ regeneration of damaged tissues [155]. The delicate balance of mechanical forces experienced by tenocytes governs tissue homeostasis and the progression of pathological conditions. Tendon homeostasis depends on mechanical loading within physiological limits, whereas excessive mechanical loading contributes to tendinopathy development. Tenocytes interpret their mechanical microenvironment through mechanotransduction, which converts mechanical stimuli into biochemical signals, thereby eliciting biological responses [156]. The mechanical characteristics of scaffolds influence the formation and maturation of engineered tendon tissue, as mechanical stress can stimulate the expression of proteoglycans, including versican. Both components are crucial for establishing the microenvironment of TDSCs and promoting matrix maturation [157]. In healthy tendons, tensile properties are primarily determined by well-aligned collagen fibrils, each with a diameter of approximately 100 nm. Human Achilles tendons demonstrate a rupture stress of up to 80 MPa and a Young’s modulus ranging from 800 to 1,000 MPa. To restore these structural and mechanical characteristics following injury, implanted scaffolds must actively guide cellular responses toward regeneration rather than fibrosis. This redirection is critical in determining the scaffold’s long-term performance and the successful formation of neo-tendon tissue that closely replicates the functionality of the native tendon. In alignment with these designs, composite hydrogel materials that integrate polymeric fibers with hydrogel matrices emulate the aligned architecture of native tendons through their oriented fibers and accelerate the recruitment of tendon progenitor cells, stimulate tenogenic differentiation, and guide cells to deposit directionally organized matrix with native tendon-like structural alignment [158]. Electrospun microfibers and methacrylated gelatin can be integrated into multilayered constructs that mimic native tendon tissue organization through photo-crosslinking of stacked scaffold sheets. Interlayer cell seeding enables the formation of cell-laden, stratified architectures that replicate the mechanical properties, structural characteristics, and cellular phenotypes of natural tendon tissue [159].

In addition to scaffold properties, importance emphasis should be placed on regulating the local biological microenvironment as a pivotal strategy to enhance tendon tissue regeneration. Tendon injuries inevitably trigger inflammation, which is accompanied by immune cell infiltration, particularly macrophages, and increased pro-inflammatory cytokine secretion. Furthermore, the production of ROS, including hydroxyl radicals, superoxide anion (O₂·^−^), and hydrogen peroxide (H_2_O_2_), is intrinsically associated with the inflammatory microenvironment following tendon injury. Excessive pro-inflammatory cytokines and ROS disrupt normal cell cycle progression and migration, induce cell death and phenotypic alterations, and severely hinder the tendon repair process. Consequently, tissue-engineered scaffolds with dual immunomodulatory and antioxidant capabilities represent a promising therapeutic strategy for remodeling the pathological cellular microenvironment within injured tendons [160].

**Fig. 1.**
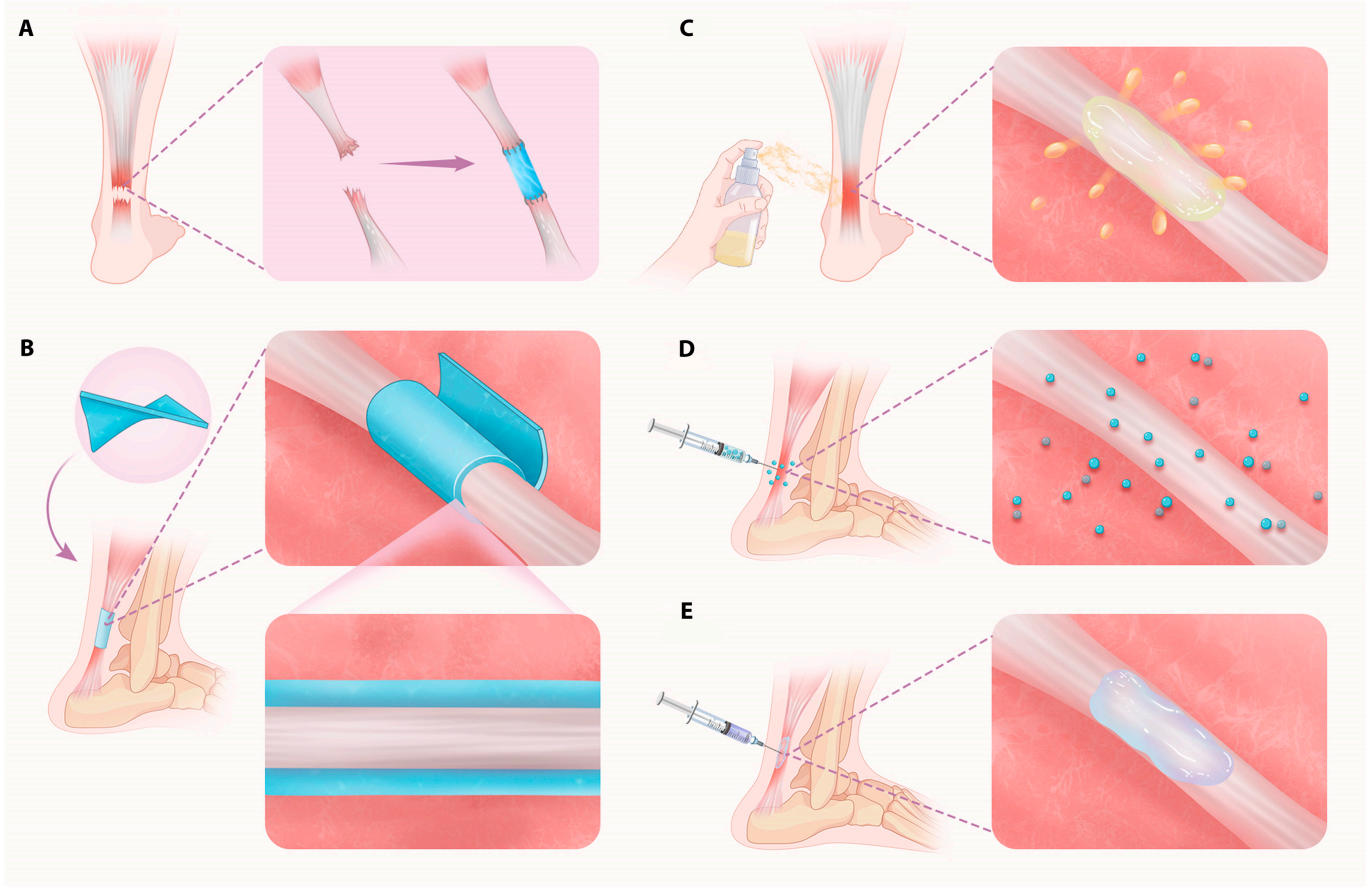
Schematics showing the contact of different hydrogel formulations with injured tendon tissue. (A) Three-dimensional porous scaffold implanted on the injured tendon surface. (B) Patch conforms to and covers a segment of the injured tendon surface. (C) Hydrogel spray gels in situ and covers the surface of the injured tendon tissue. (D) Hydrogel microspheres are dispersed onto the surface of the injured tendon. (E) Injectable hydrogel infiltrates the injured tendon and undergoes in situ gelation.

In summary, hydrogel scaffolds have become fundamental materials in tissue engineering and regenerative medicine, attributed to their exceptional biocompatibility and structural biomimicry. These properties enable precise replication of the ECM, providing a supportive platform that closely replicates the in vivo environment (Fig. 1A). Hydrogel scaffolds offer a unique combination of mechanical strength, inherent flexibility, and compliance, enabling seamless adaptation to complex physiological environments. Furthermore, their biodegradable and bioresorbable characteristics allow for natural decomposition within the body, eliminating the necessity for secondary surgical removal and minimizing patient discomfort and associated clinical risks. These advanced characteristics, encompassing tunable porosity for nutrient diffusion, cell-instructive surface topography, and on-demand degradation kinetics, establish hydrogel scaffolds as revolutionary platforms in tendon regeneration engineering.

### Hydrogel patches

Adhesions between the tendon and surrounding tissues are the most severe complication of tendon repair, primarily caused by the chemotaxis of extrinsic fibroblasts [161]. Furthermore, evidence indicates that external repair mediated by cells derived from the surrounding tendon sheath and synovium is a principal contributor to postoperative tendon adhesions, and the correlation between inflammatory responses and adhesion formation is of critical important [73]. The hydrogel patch adheres effectively to diverse tissue surfaces, emulating the functional properties of ECM by permitting the diffusion of cells, cytokines, oxygen, and other components while simultaneously acting as a physical barrier. This mechanism mitigates exogenous tendon repair and inflammatory responses, facilitating functional tendon regeneration (Fig. 1B).

The hydrogel patch fabricated through a one-step dialysis process employs PVA and gelatin as the foundational components of its physical barrier architecture. The interplay between the flexibility of gelatin and the stiffness of PVA polymers, combined with in situ-formed hydrophobic aggregates and an extensive network of hydrogen bonds, synergistically imparts the hydrogel patch with high toughness, exceptional strength, and a low elastic modulus, along with excellent fatigue resistance and self-recovery capabilities. Moreover, the hydrophobic bio-multifunctionality of benzyl isothiocyanate facilitates self-assembly onto the hydrogel patch during the sol-gel transition, enabling it to remodel the inflammatory microenvironment through synergistic antibacterial, antioxidant, and anti-inflammatory effects. This multifunctional capability positions it as a promising candidate for preventing postoperative adhesions and infections [162].

Persistent release of pro-inflammatory cytokines by M1 macrophages induces cellular apoptosis and disordered collagen deposition, whereas M2 macrophages promote fibroblast proliferation, coordinate anti-inflammatory responses, stabilize angiogenesis, and facilitate ECM remodeling. In response to these challenges, a dual-crosslinked hydrogel patch has been engineered using methacryloyl-substituted *Bletilla striata* polysaccharide and gelatin via UV-initiated polymerization. This advanced patch efficiently modulates M1-to-M2 macrophage polarization without needing exogenous cytokine supplementation, enhancing fibroblast proliferation and migration while expediting vascular network formation [152]. Additionally, a patch fabricated by impregnating plain-woven polyethylene terephthalate mesh with decellularized matrix gel and subsequently subjected to lyophilization exhibits reliable mechanical properties and excellent biocompatibility. Its loosely porous structure effectively promotes tendon cell proliferation, migration, and differentiation while enhancing histological and biomechanical outcomes in rabbit models of rotator cuff tear [163].

As an advanced wound dressing material, the hydrogel patch functions as a physical barrier with strong tissue adhesion capability and integrates superior biocompatibility, anti-swelling properties, bionic mechanical characteristics, and multifunctional biological activity. Its distinct material advantages are exemplified by a 3D porous sponge structure, sustained water retention exceeding 90% content, and stable performance in loading active substances. These features position it as an optimal carrier system for stem cell therapy and underscore its substantial potential in wound repair applications. This system typically employs crosslinked networks of natural/synthetic polymer as the matrix framework, incorporating therapeutic components to construct composite systems that include but are not limited to functional cells (such as MSCs), bioactive macromolecules (including proteins and nucleic acids), and specific growth factors. This advanced drug delivery system extends the bioactivity duration of therapeutic factors at the wound site through sustained release mechanisms, facilitating precise temporal regulation of therapeutic interventions.

### Hydrogel spray

Sprayable hydrogels offer advantages in tissue engineering due to their superior mixing capabilities, adjustable spraying parameters, and portability. These properties facilitate rapid and uniform coverage of irregularly damaged tissues in a liquid state, enabling deep tissue penetration and effectively functioning as an anti-adhesion barrier. This functionality is critical for preventing postoperative complications such as tissue infection and adhesion. In contrast to preformed hydrogels, in situ-forming hydrogels have recently emerged as highly promising in biomedical materials. Their gelation mechanisms are triggered through solvent exchange, UV irradiation, ionic crosslinking, pH variation, and temperature modulation. These hydrogels can be directly administered into the body via spraying, offering multiple advantages such as minimally invasive application, procedural simplicity, high efficiency in cell encapsulation, and effective drug loading. The hydrogel precursor system exhibits shear-thinning behavior under shear stress during spraying, subsequently undergoing immediate transformation into solid hydrogels at the implantation site by forming physical and/or chemical crosslinking networks. Hydrogels, recognized as a quintessential biocompatible material, are extensively utilized across biomedical domains, including drug delivery, tissue engineering, and cell therapy [164,165] (Fig. 1C).

Postoperative adhesions may result in pain, functional impairment, and the necessity for secondary surgical interventions. Various biodegradable anti-adhesion barriers have been investigated to mitigate postoperative adhesions; however, most available materials exhibit unsatisfactory therapeutic efficacy due to their inconvenient application. Hydrogels derived from natural polymers are preferred due to their excellent biocompatibility and degradability, effectively overcoming critical clinical application challenges. In contrast to other hydrogel systems, sprayable hydrogels confer distinct advantages, as the formulated biomaterials can be applied directly via spraying, enabling effective coverage of intricate tissue architectures and facilitating compatibility with minimally invasive interventions. Utilizing a specialized syringe-based atomized spraying technique, these materials establish a protective barrier over wounds, inhibiting bacterial growth while combining portability with rapid in situ film-forming capabilities. Their immediate film-forming characteristics facilitate tight integration with surrounding tissues, ensuring excellent flexibility and enhancing the healing process, particularly in the case of irregular tissue injuries. This approach holds potential for preventing postoperative tendon adhesions and infections while promoting tendon tissue regeneration and structural reconstruction. The degradable, sprayable glycyrrhetinic acid (GA) hydrogel derived from natural GA is fabricated through a simple heating and cooling process that eliminates additional chemical crosslinking agents needed to prevent postoperative adhesions. This carrier-free, sprayable GA hydrogel enables the sustained release of GA to suppress inflammatory responses and functions as a physical anti-adhesion barrier, reducing collagen deposition and fibrosis [164,166].

### Hydrogel MSs

Due to their biocompatibility, fluidity, porous structure, and large specific surface area, hydrogel MSs provide excellent physical and biological conditions conducive to tendon healing and regeneration. Hydrogel MSs, a specialized subclass of hydrogels ranging in size from micrometers to millimeters, retain the essential properties of conventional hydrogels while providing distinct functional advantages. Their small volume facilitates in vitro preparation and crosslinking prior to syringe-mediated in vivo injection, while subsequent in situ crosslinking exhibits improved structural stability. Consequently, injectable suspensions incorporating these MSs exhibit exceptional fluidity and prolonged in vivo retention time, making them particularly suitable for therapeutic delivery across various tissue types. Furthermore, since hydrogel MSs can be entirely fabricated and crosslinked in vitro, they permit precise customization and processing to address specific requirements, thereby facilitating the construction of complex architectures with multifunctional properties [167].

Tissue regeneration is a relatively protracted and coordinated process, making the controlled and sustained paracrine activity of stem cells essential for optimizing therapeutic outcomes. Hydrogel MSs, fabricated using microfluidic technology, exhibit relatively small sizes and high monodispersity, facilitating minimally invasive needle delivery. By providing an expanded specific surface area, these MSs enhance oxygen and nutrient exchange between cells and the extracellular microenvironment, thereby preserving cellular viability while shielding cells from direct exposure to harsh conditions that could compromise cellular metabolism. The small size and biodegradability of hydrogel MSs promote cell infiltration and aggregation, thereby enhancing intercellular ECM deposition and facilitating cell–cell interactions. The porous structure and superior mechanical properties of methacrylated gelatin hydrogel MSs confer excellent cytocompatibility, which improves stem cell survival and engraftment rates by fostering cell adhesion. Exogenous stem cells exhibit strong adhesion to and proliferation on these hydrogel MSs, reinforcing intercellular matrix deposition and enhancing cell–cell communication while amplifying paracrine effects [153]. The high water content of hydrogel MSs enables their mechanical properties to closely replicate those of natural tissues, thereby alleviating mechanical stress after implantation [168].

Hydrogel MSs exhibit improvements in fluidity and flexibility, facilitating their seamless dispersion and subsequent lubrication enhancement. The micron-scale spherical structure of these MSs imparts exceptional physical rolling capability, which optimizes their mobility and local tissue distribution, enabling them to penetrate deep pathological tissues via syringe needles and effectively conform to irregular tissue cavities [169] (Fig. 1D). Furthermore, bioactive substances (such as growth factors, cells, and drugs) can be encapsulated within hydrogel MSs through electrostatic interactions, forming biocompatible biological or functional materials with excellent performance [170].

### Injectable hydrogels

Injectable hydrogels exhibit reversible fluid–gel state transitions, enabling perfect conformation to tissue contours of any shape, providing lubrication and mechanical support while effectively preventing tendon adhesion. Furthermore, they are carriers for bioactive substances that enhance cellular motility and maintain viability. Notably, they offer a targeted, minimally invasive implantation approach, which has garnered preference from patients. Biomaterials can be injected into the body in liquid form, subsequently undergoing in situ solidification and possessing the necessary physicochemical properties for in vivo injection applications (Fig. 1E). Commonly utilized materials for forming injectable hydrogels include CS, collagen or gelatin, alginate (ALG), HA, heparin, chondroitin sulfate, PEG, and PVA. Covalent crosslinking, weak secondary interactions, photo-crosslinking, Michael addition reactions, and click chemistry can all be utilized to fabricate ion-sensitive, pH-responsive, or thermosensitive injectable hydrogels with precisely tunable properties. Injectable hydrogel mechanisms can be classified into 3 main categories: in situ gelling liquids, which are solutions or liquids that typically flow but solidify into gels when injected into the body. Injectable particles maintain their injection capability when immersed in liquid phases, with particle sizes potentially ranging from the nano- to micro-scale or macro-scale, depending on the desired outcomes [171]. Moreover, these hydrogels exhibit shear-thinning behavior (a reduction in viscosity under external force) while rapidly restoring their network structure upon force removal [172], making them ideal candidates for minimally invasive injectable biomaterials.

Conventional direct cell injection often results in cell loss due to dispersion into surrounding tissues or cell death induced by shear stress during injection. Consequently, incorporating cells, bioactive factors, or drugs into injectable hydrogels offers an optimal solution for patients who prefer minimally invasive therapies. Injecting hydrogels into target sites induces no damage to the organism while preserving the structural and functional integrity of the target tissue after injection. These injectable hydrogels function as versatile carriers for drugs and cells, enabling targeted delivery through minimally invasive procedures [173]. For instance, this bioactive agent can be effectively delivered to injured tendons using minimally invasive procedures. The injectable particulate HA hydrogel is specifically engineered to deliver fibromodulin (Fmod), a bioactive ECM component that enhances tenocyte motility while optimizing surrounding ECM organization to promote tendon healing [174]. Bioactive glass incorporated with SA forms an injectable hydrogel system in which gradual dissolution releases Ca^2+^ that autonomously trigger ALG crosslinking, enabling in situ gelation without external stimuli. This system improves tendon repair by enhancing ultimate tensile load, failure stress, and tensile modulus while promoting angiogenesis and facilitating macrophage polarization from pro-inflammatory M1 to anti-inflammatory M2 phenotype without ectopic ossification. Specifically engineered to augment tendon regeneration following surgical repair, the hydrogel adapts to irregular defect geometries, mitigates adverse effects of substantial muscle retraction, shortens operative duration, minimizes surgery-associated pain and scar formation, accelerates postoperative recovery, and lowers healthcare expenditures [175].

## Formulation Design of Hydrogel Platforms for Tendinopathy Management

A rational hydrogel formulation design was developed through systematic investigation of the physicochemical properties of constituent materials and modulation of their molecular structures. Optimizing network architectures and employing dynamic crosslinking mechanisms achieved significant enhancements in biocompatibility, mechanical performance, and biological functionality. These enhancements were subsequently validated by favorable outcomes in both in vitro cellular experiments and in vivo animal models (Table 1).

**Table 1.** Formulation design of hydrogel platforms for tendinopathy management

Hydrogel platforms	Material	Crosslinking methods	Material features	Outcome	Animal models or case study	References
Hydrogel scaffolds	MHA-sEVs	Photo-crosslinking/Ca^2+^ crosslinking	Facilitate nutrient exchange and metabolite removal; promote cell infiltration and tissue growth	Enhance M2 macrophage polarization with reduced pro-inflammatory cytokine secretion; increase COL I deposition and decreased COL III content; promote tendon maturation and superior biomechanical strength	Osteoporotic RCR rat model	[176]
	PCL/MLT-ALG	Ca^2+^ crosslinking	Demonstrate excellent biocompatibility while promoting cell proliferation and differentiation	Promote TDSCs survival, proliferation, and tenogenic differentiation; reduce macrophage infiltration; activate Nrf-2/HO-1 antioxidative pathway to decrease ROS production	Surgically induced Achilles tendon defect rat model	[177]
	CS	–	Conducive to cell inward growth and nutrient penetration; provide mechanical support and physical barriers	Promote tenogenic differentiation of TSPCs; reduce tendon adhesion and improve repair quality	Surgically induced Achilles tendon defect rat model	[178]
	TenoGlide	Hydrogen bond crosslinking/hydrophobic interaction crosslinking	Exhibit good cell compatibility and a loose, spongy microstructure	Reduce tendon adhesion and increase tendon gliding	Case of chronic peroneus brevis tendon injury	[179,180]
	GelMA	Photo-crosslinking	Facilitate material exchange; provide physical support for cell adhesion and migration	Promote tendon and cartilage formation, enhance mechanical properties, and improve tendon-to-bone healing	SSP tendon rupture New Zealand white rabbit model	[181]
	Fibrin/HA/PLGA	Enzyme-mediated crosslinking	Provide cell with adhesion and growth sites and a mechanical microenvironment	Up-regulate tendon-related gene expression at the mRNA level in hBMSCs and induce tenogenic differentiation	–	[182]
	GG/CR/CMC/PVA	Ca^2+^ crosslinking	Exhibit biocompatible, degradability, swellability, and mechanical robustness	Accelerate tendon regeneration by promoting fibrous tissue formation, angiogenesis, and collagen deposition	Surgically induced Achilles tendon defect rat model	[183]
	GelMA/PRP	Photo-crosslinking	Exhibit excellent cytocompatibility	Enhance tenocyte viability; activate NF-κB/p38-MAPK signaling to promote angiogenesis	–	[184]
	PEG-4MAL	Michael addition reaction	Exhibit excellent biocompatibility; serve as a carrier for ECM constructs	Improve cell morphology; reduce COL III content; enhance matrix alignment; increase tendon stiffness; promote tendon healing	Surgically induced patellar tendon defect mice model	[185]
	SA/Fibrinogen	Ca^2+^ crosslinking	Promote cell adhesion and growth	Facilitate the formation of tendon-like ECM by promoting Col I and III secretion in NIH 3T3 fibroblasts	–	[186]
	GBTH	Photo-crosslinking	Exhibit excellent biocompatibility, high strength/toughness, and degradability	Enhance tendon mesenchymal stem cell differentiation; promote increased collagen fiber quantity with well-aligned organization and high mechanical strength	Surgically induced tendon defect rabbit model	[187]
Hydrogel patches	XG/GG/HA	EDC/NHS crosslinking	Exhibit high water content, slow degradation rate, excellent mechanical properties, and good cytocompatibility	Prevent tendon adhesion and promote healing while maintaining mechanical strength	Surgically induced Achilles tendon rupture rat model	[188]
	ALG/PAAM	Ca^2+^ crosslinking/covalent bond crosslinking	Exhibit strong adhesion to multiple tendons, low friction on nonadhesive side for tendon gliding, and high drug loading with controlled release	Modulate cytokine secretion; recruit TSCs; promote macrophage M2 polarization reduce inflammation	Surgically induced patellar tendon defect, rotator cuff injury, and Achilles tendon rupture rat model	[189]
	PZBA-EGCG-ALC Janus	Photo-crosslinking	Possess high tensile strength, elongation at break, and asymmetric adhesion	Prevent postoperative adhesions; promote tendon healing by providing mechanical support, acting as a physical barrier, and reducing early inflammation	Surgically induced Achilles tendon adhesion rat model	[190]
	Thiol-gelatin	Coordination bond crosslinking	Exhibit excellent biocompatibility and antibacterial properties; achieve controlled ion release	Promote tenocyte proliferation; down-regulate Col III expression; promote tendon–bone interface regeneration	Surgically induced RCT rat model	[191]
	PEG-PLV	–	Feature biocompatibility and degradability; load and sustain bFGF release	Prevent tendon adhesion; reduce inflammatory cell infiltration; promote tendon healing	Surgically induced Achilles tendon injury rat model	[192]
	PEG/GA/LA	Self-assembling crosslinking	Exhibit good lubricity, anti-cell adhesion, and biocompatibility.	Reduce fibrosis and vascularization; decrease peritendinous adhesions; improve Achilles tendon mobility	Surgically induced Achilles tendon adhesion rat model	[193]
Hydrogel spray	Cur@ZIF-8@CeO_2_@HAMA	Photo-crosslinking	Photosets within seconds forming hydrogel films; exhibit porous structure, facilitating drug loading and release	Down-regulate NF-κB/IL-17 signaling to reduce macrophage inflammatory cytokine/chemokine release and restore homeostasis; inhibit THO formation at the injury site	Surgically induced THO mice and rat model	[195]
Hydrogel MSs	siRNA@MS@HA/PCL	Hydrazone crosslinking	Exhibit self-healing capability and MMP-2 responsiveness	Silence Smad3 to inhibit fibroblast proliferation; reduces inflammation and prevents peritendinous adhesion formation	Surgically induced tendon injury rat model	[198]
	GM@HDC@HGF	Photo-crosslinking	Possess porous structure; facilitate drug loading and release	Alleviate inflammation and oxidative stress to promote tendon regeneration and repair	Collagenase I induced AT rat model	[199]
	GelMA/SF@TPCA-1	Photo-crosslinking	Provide a hydrated microenvironment for cell survival; enable drug loading and release	Promote M2 macrophage polarization; inhibit ectopic ossification; enhance tendon regeneration and functional recovery	Surgically induced patellar tendon defect and fracture rat model	[200]
	GM@PDA&SHED-Exo	Photo-crosslinking	Exhibit excellent biocompatibility and porous architecture; facilitate exosome encapsulation and controlled release	Restore TSPCs self-renewal and differentiation capacity; inhibit tendon pathology in mice and ectopic ossification in aged rats, while promoting tendon repair	Tendon degeneration natural aging mice model; collagenase I-induced tendinopathy mice model; surgically induced Achilles tendon rupture rat model	[201]
	PH/GMs@bFGF&PDA	Phenylboronate ester crosslinking	Conforms to complex tendon defects; exhibit self-healing capability and strongly adhesive	Reduce inflammatory cell infiltration and promotes tendon wound healing	Surgically induced acute and chronic tendon injury New Zealand rabbit model	[202]
Injectable hydrogels	PCNP	Hydrophobic interaction crosslinking	Encapsulate and sustained release drug	Modulate the immune microenvironment; promote collagen synthesis to facilitate Achilles tendon repair and functional recovery	Collagenase induced Achilles tendon injury rat model	[205]
	SIS@Gas	–	Possess biocompatibility, water imbibition, injectable, porous structure; wrap and promote the long-term and slow release of drugs	Suppress inflammation; promote Col I expression; suppress Col III; modulate SCX/TNMD expression	Collagenase I induced AT rat model	[206]
	HA@EGCG	Hydrazone crosslinking	Achieve drug loading and continuous release	Improve collagen fiber structure; reduce cellularity and vascularization, and suppress the gene expression of the Pparg	Collagenase I induced AT rat model	[207]
	GDF5@DPH	Self-assembling crosslinking	Conducive to the adhesion and growth of cell	Suppress inflammation; enhance regeneration and repair of injured tendons; improve mechanical properties	Surgically induced Achilles tendon defect New Zealand white rabbit model	[208]
	Fibrinogen/BMSCs-Exos	Enzyme-mediated crosslinking	Encapsulate and release BMSCs-Exos	Promote TSPCs proliferation and COL I deposition; enhance tendon mechanical properties	Surgically induced patellar tendon defect rat model	[209]
	SA/Collagen	Ca^2+^ crosslinking	Possess biodegradability; enable ADSCs loading and controlled release	Enhance angiogenesis and tissue repair	Surgically induced Achilles transection injury rat model	[210]
	PE-IAH	Secondary amine bond crosslinking	Generate a weak microcurrent to stimulate cell proliferation and differentiation	Generate sustained electrical signals that induce accelerated proliferation and oriented alignment of TSCs; prevent the formation of adhesions	Surgically induced Achilles tendon rupture rat model	[211]
	H-Exos-gel	–	Possess biodegradability	Promote M2 macrophage polarization while suppressing M1 polarization; promote structural regeneration and functional recovery of tendons	Surgically induced Achilles tendon rupture rat model	[212]
	MDP@Oxo-M@4-PPBP	Self-assembling crosslinking	Possess biocompatibility; realize the encapsulation and release of small-molecule drugs	Promote M2 macrophage polarization and IL-10/TIMP-3 expression; improve tendon healing structure and mechanical properties	Surgically induced patellar tendon transection injury rat model	[213]
	CP@SiO_2_	Self-assembling crosslinking	Promote cell migration and growth	Modulate inflammatory response; promote tissue repair; improve tendon function	Surgically induced tendon rupture healing rat model	[204]
	tdECM@TDSCs-Exos	–	Conducive to the exchange of nutrients and fluids	Regulate macrophage polarization via PI3K-Akt and MAPK signaling pathways; promote tendon repair and enhance mechanical properties	Collagenase I induced AT New Zealand white rabbit model	[214]
	Cur&Mg-QCS/PF	Schiff base crosslinking/metal-coordination crosslinking	Feature self-healing and porosity; provides mechanical support and facilitate mass transfer	Modulate the inflammatory microenvironment to promote rotator cuff tendon-to-bone healing	Surgically induced RCT rat model	[215]
	CS/β-glycerophosphate	Electrostatic interaction crosslinking/hydrogen bond crosslinking	Possess a nontoxic, biodegradability and drug release of duplex mode	Block the Rac1 signaling pathway to suppress pathological osteogenic differentiation of TSPCs improve tendon tissue quality; reduce inflammation and angiogenesis	Collagenase I induced Achilles tendon degeneration rat model	[216]
	RADA+TSPCs	Self-assembling crosslinking	Possess good biocompatibility, providing a suitable survival microenvironment for TSPCs	Accelerate tendon functional recovery and reduce ectopic ossification	Surgically induced patellar tendon defect rat model	[217]
	TSPCs-Gel	Self-assembling crosslinking	Provide nutrients for cell through a microporous structure; resist external shear forces with a rigid structure	Up-regulate the expression of Col I and TNMD while down-regulating Col III; alleviate peritendinous inflammation and swelling; restore mechanical properties	Collagenase I induced AT rat model	[220]
	ACHP-Gel	Self-assembling crosslinking	Enhance ACHP solubility through porous microstructure; possess excellent degradation capability	Down-regulate inflammation-related genes and ECM remodeling-related genes; up-regulating tendon differentiation genes	Collagenase I induced AT rat model	[221]

ACHP, 2-amino-6-[2-(cyclopropylmethoxy)-6-hydroxyphenyl]-4-(4-piperidinyl)-3-pyridine carbonitrile; ADSCs, adipose-derived stem cells; ALC, acrylic acid/lauryl methacrylate/hexadecyl trimethyl ammonium bromide; ALG, sodium alginate; AT, Achilles tendinopathy; BMSCs, mesenchymal stem cells; bFGF, basic fibroblast growth factor; CeO_2_, ceric oxide; Col I, type I collagen; Col III, type III collagen; CP@SiO_2_, comprising puerarin, chitosan, and mesoporous silica nanoparticles; CS, chitosan; Cur, curcumin; DPH, dipeptide hydrogel; ECM, extracellular matrix; EDC, 1-ethyl-3-(3-dimethylaminopropyl)-carbodiimide; EGCG, epigallocatechin gallate; Exos, exosomes; Exos-gel, comprising human umbilical vein endothelial cell-derived exosomes, chitosan, and hydroxyethyl cellulose; Gas, gastrodin; GBTH, gelatin-based tough hydrogel; GDF5, growth differentiation factor 5; GelMA, gelatin methacryloyl; GM, gelatin methacryloyl microspheres; GMs, gelatin microspheres; HA, hyaluronic acid; HAMA, methacrylate catalyzed HA; HDC, heparin–dopamine conjugate; HGF, hepatocyte growth factor; HO-1, heme oxygenase-1; hBMSCs, human bone marrow mesenchymal stem cells; IL, interleukin; MDP, multidomain peptide; Mg, magnesium ion; MHA-sEVs, sodium alginate/hyaluronic acid/small extracellular vesicles from adipose-derived stem cells; MLT, melatonin; MMP-2, matrix metalloproteinase-2; MSs, microspheres; NF-κB, nuclear factor-kappa B; NHS, N-hydroxysuccinimide; Nrf-2, nuclear factor erythroid-2-related factor 2; Oxo-M, oxotremorine M; PCL, polycaprolactone; PCNP, poly(organophosphazene)(PPZ)-celecoxib(CXB) nanoparticles; PDA, polydopamine; PE-IAH, piezoelectric injectable anti-adhesive hydrogel; PF, aldehyde groups from benzaldehyde-terminated PluronicF127 (PF127-CHO) polymer; PH, polyvinyl alcohol (PVA)-aminophenylboronic acid-modified hyaluronic acid (BA-HA) hydrogels; PI3K-Akt, phosphatidylinositol 3-kinase-protein kinase B; PLGA, poly-lactic-co-glycolic acid; Pparg, peroxisome proliferator activated receptor gamma; PRP, platelet-rich plasma; PZBA, poly[(N,N-dimethylethylenediamine)-g-(N,N,N,Ndimethylaminoethyl-p-methylphenylboronic acid ammonium bromide-g-(N,N,N,N-dimethylaminoethyl ethyl acetate ammonium bromide)]; MAPK, mitogen-activated protein kinase; QCS, quaternized chitosan; RADA, Ac-(RADA)_4_-CONH_2_; Rac1, Ras-related C3 botulinum toxin substrate 1; RCR, rotator cuff repair; RCT, rotator cuff tear; ROS, reactive oxygen species; SA, sodium alginate; SCX, scleraxis; SF, silk fibroin; SHED, stem cells from human exfoliated deciduous teeth; SIS, small intestinal submucosa; SSP, supraspinatus; siRNA, small interfering RNA; TDSCs, tendon-derived stem cells; THO, traumatic heterotopic ossification; TIMP-3, metalloproteinases-3; TNMD, tenomodulin; TSCs, tendon stem cells; TSPCs, tendon stem/progenitor cells; tdECM, tendon-derived decellularized extracellular matrix; ZIF-8, zeolitic imidazolate framework-8; 4-PPBP, 4-phenyl-1-(4-phenylbutyl) piperidine

### Hydrogel scaffolds

Hydrogels are widely used in tendon repair and regeneration as a distinctive polymeric material possessing 3D network structures. Their porous architecture, high water content, and exceptional resemblance to native cellular and tissue microenvironments endow hydrogel-based scaffolds with outstanding biocompatibility. These characteristics enable them to establish localized microenvironments that regulate cellular growth and differentiation, promote tendon tissue formation, and serve effectively as anti-adhesion barriers to suppress scar tissue development. Currently, research on hydrogel scaffold materials for the treatment of tendinopathy focuses on several critical therapeutic strategies, including stem cell-based therapy, anti-inflammatory, antioxidant, anti-adhesion, and antibacterial effects, as well as biomimetic scaffolds featuring micro-topological structures.

**Fig. 2.**
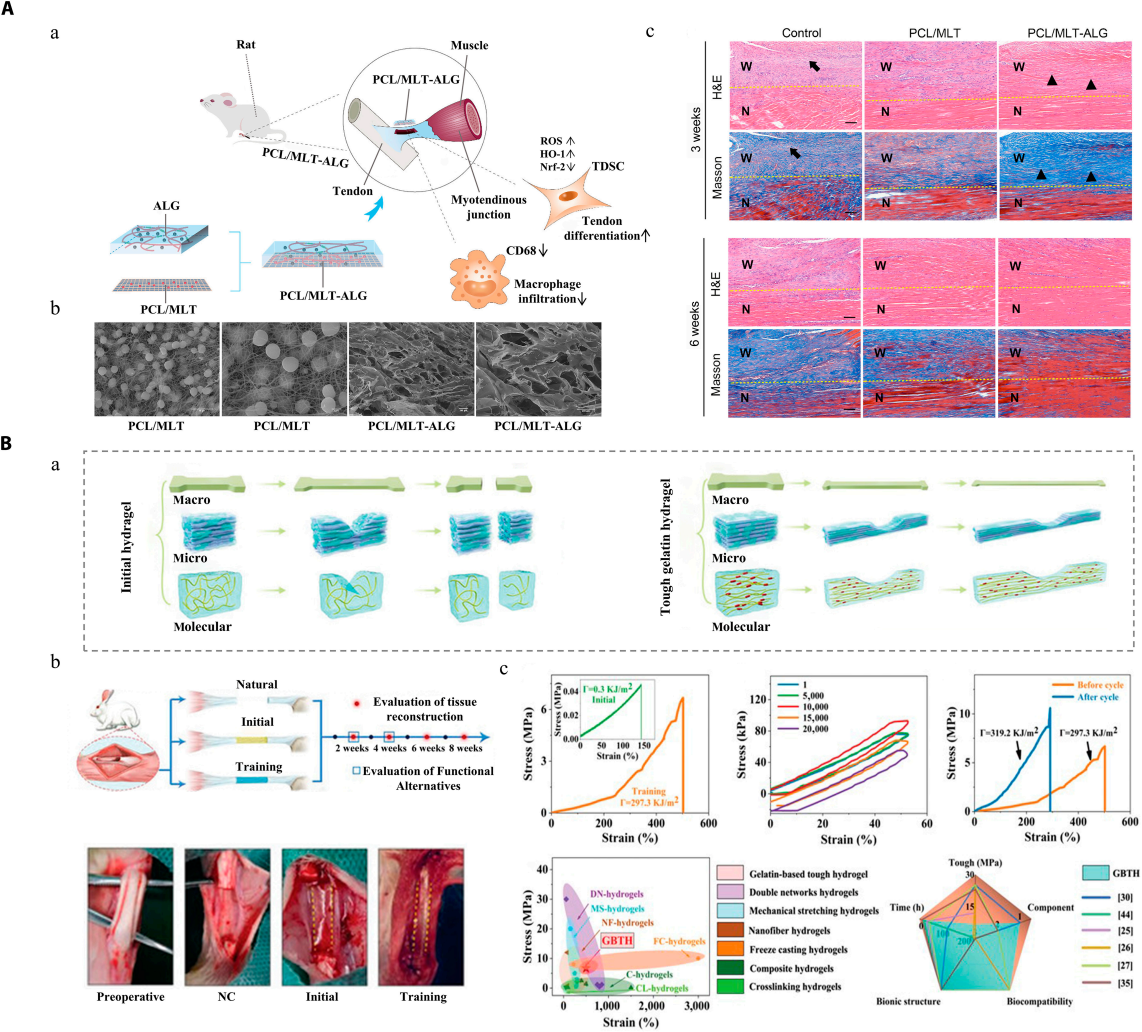
Design and fabrication of hydrogel scaffolds for tendinopathy treatment. (A) MLT-loaded PCL/ALG composite scaffold facilitate tendon regeneration. (a) Schematic illustration. (b) Characterization of PCL/MLT electrospun membrane and PCL/MLT-ALG scaffolds. (c) Histological staining and tendon healing evaluation at 3 and 6 weeks post-operation. Reprinted from Yao et al. [177]. Copyright 2022, the authors. (B) GBTH promotes tendon differentiation and tissue regeneration. (a) Schematic diagram of the mechanism of hydrogel intensification. (b) In vivo tendon repair experiments. (c) Properties of gelatin-based tough hydrogel. Reprinted from Yuan et al. [187]. Copyright 2023, the authors. ALG, sodium alginate; H&E, hematoxylin and eosin; HO-1, heme oxygenase-1; MLT, melatonin; Nrf-2, nuclear factor erythroid-2-related factor 2; PCL/MLT-ALG, PCL loaded with MLT membrane sodium alginate hydrogel; PCL, polycaprolactone; ROS, reactive oxygen species.

Song et al. [176] developed a macroporous hydrogel scaffold incorporating aligned microfibrous architecture using SA, HA, and small extracellular vesicles derived from adipose-derived stem cells (ADSCs). The macroporous structure of the scaffold enhances cellular infiltration and tissue integration capabilities, while the aligned microfibrous gel architecture effectively promotes the tenogenic differentiation of TDSCs. Similarly, a PCL/melatonin (MLT)/ALG scaffold composed of PCL, MLT, and sodium ALG was designed and fabricated (Fig. 2A, a and b). This composite scaffold enhanced the proliferation, viability, and tenogenic differentiation of TDSCs while markedly up-regulating the expression of tendon-specific markers, including Col Iα1, decorin, and TNMD and elevating antioxidant marker expression. In vivo studies validated the PCL/MLT/ALG scaffold’s capacity to promote tendon regeneration (Fig. 2A, c) [177]. The CS scaffold’s sponge layer, characterized by its large surface area and high porosity, facilitates cellular ingrowth and nutrient permeation, while the dense CS membrane layer imparts mechanical strength and functions as a physical barrier to effectively prevent postoperative adhesion formation. Rat TSPCs cultured on this asymmetric CS scaffold demonstrated excellent adhesion, spreading, and preserved normal morphology, along with up-regulation of tendon-specific genes and proteins such as SCX and TNMD, promoting tenogenic differentiation [178]. Interestingly, TenoGlide is a Col I and GAG-based scaffold clinically used in tendon therapy. When applied as a wrap around repaired tendons, it reduces the risk of peritendinous adhesions and optimizes the gliding function of tendon transfers [179,180].

The porous architecture of the hydrogel establishes a robust framework for the loading and controlled delivery of therapeutic agents, cellular components, and bioactive substances. For instance, a GelMA-based hydrogel scaffold incorporating kartogenin enables sustained drug delivery and effectively enhances the differentiation of bone marrow mesenchymal stem cells (BMSCs) into chondrocytes. In a rotator cuff tear model, this scaffold successfully repaired tendon injuries through bone marrow stimulation [181]. Cytokines are essential regulators in the tendon healing process. A hydrogel scaffold fabricated using PLGA microcarriers and HA enables the controlled release of human growth differentiation factor 5 (hGDF-5). The released hGDF-5 acts synergistically with cyclic mechanical strain to up-regulate the expression of genes associated with tendon differentiation in human BMSCs, thereby directing their differentiation toward the tenocyte lineage [182]. A hydrogel scaffold, formulated with gellan gum as the primary polymer and crosslinked with κ-carrageenan, CMC, and PVA in the presence of Ca^2+^, exhibits biphase drug release kinetics and ensures the continuous drug release of rosuvastatin calcium. In the rat model of Achilles tendon injury, the scaffold enhances fibrous tissue formation, stimulates angiogenesis, and elevates collagen expression, accelerating tendon tissue regeneration [183]. Del Amo et al. [184] employed 3D bioprinting technology to fabricate platelet-rich plasma (PRP) scaffold grafts, which enhanced the tendon cell vitality, promoted the migration of endothelial cells, and increased vascular area and connection density. Under inflammatory conditions, PRP grafts demonstrated elevated activity of the VEGF signaling pathway while simultaneously activating the NF-κB and the p38-MAPK signaling pathways to promote angiogenesis.

Achieving increased mechanical strength represents a primary objective of tendon repair; therefore, recovering tendon structure and mechanical functionality is critical. A scaffold hydrogel synthesized using tendon-derived ECM, PEG, and maleimide enhanced the matrix arrangement and tendon stiffness, reduced the expression of Col III in the healed tendon, improved cellular morphology, and more closely resembled the characteristics of healthy tenocytes [185]. Additionally, the hydrogel scaffold developed by Volpi et al. [186] using fibrinogen and ALG can mimic the mechanical properties of native muscle and tendon tissues. NIH 3T3 fibroblasts orient along the microfiber axis, enhancing their responsiveness to external mechanical stimuli and activating relevant signaling pathways. A gelatin-based tough hydrogel (GBTH) fabricated using gelatin as the matrix exhibits excellent mechanical properties, including a high fatigue threshold and remarkable toughness (Fig. 2B, a and c). This hydrogel can transmit muscle contraction-mimicking stimuli to promote tenogenic differentiation of tendon mesenchymal stem cells. In vivo experiments reveal that GBTH facilitates morphological and functional recovery of repaired tissue, achieving a state nearly indistinguishable from normal tissue within 8 weeks (Fig. 2B, b) [187].

### Hydrogel patches

The hydrogel patch is a physical barrier that adheres to various tissue surfaces and mimics ECM functions. It permits the permeation of cells, cytokines, oxygen, and other components while increasing direct surface contact and minimizing diffusion barriers. This design facilitates targeted drug delivery from the hydrogel drug reservoir to intended tissues rather than surrounding tissues, thereby reducing exogenous tendon repair and inflammation while decreasing adhesion risks and ultimately promoting functional tendon restoration. Kuo et al. [188] developed a structurally robust hydrogel membrane by crosslinking xanthan gum, gellan gum, and HA using 1-ethyl-3-(3-dimethylaminopropyl) carbodiimide and N-hydroxysuccinimide. This membrane possesses high water content, rapid swelling equilibrium, excellent cytocompatibility, and a slow degradation rate. In animal studies, it effectively prevented Achilles tendon adhesion, enhanced tendon healing, and preserved mechanical strength throughout the healing process. In addition to adhesive properties, hydrogel patches are also required to exhibit certain lubricating characteristics. To meet these requirements, a mechanically resilient hydrogel patch was fabricated using SA, acrylamide, and UP CS. The patch demonstrated rapid and robust adhesion to live mouse patellar tendons, supraspinatus tendons, and Achilles tendons, maintaining stable performance in vivo. Its nonadhesive surface exhibited a low friction coefficient, facilitating tendon gliding and mitigating the risk of fibrotic scar formation. Furthermore, through the loading and sustained release of triamcinolone acetonide, the patch effectively alleviated inflammation, modulated chemokine secretion, recruited TSPCs, and promoted macrophage polarization toward the M2 phenotype. These properties enhanced healing in a rat Achilles’ tendon rupture model while minimizing scar tissue formation [189]. Similarly, the Janus hydrogel exhibits strong adhesion on its bottom surface while maintaining a adhesion difference between its 2 sides. This design allows the patch to deliver robust mechanical support through firm adhesion, effectively preventing postoperative adhesion. The hydrogel demonstrates excellent scavenging capabilities of ROS and reactive nitrogen species, which help mitigate oxidative damage. Additionally, it alleviates early-stage inflammatory responses in tendons and accelerates the tendon-healing process [190]. The gradient bimetallic hydrogel patch integrates exceptional mechanical properties with anti-scarring functionality. Loading and controlling the release of zinc ions (Zn^2+^) up-regulates the expression of SCX protein in tenocytes while simultaneously enhancing Col I production and reducing scar tissue-associated Col III expression. This dual mechanism effectively promotes tendon regeneration [191].

**Fig. 3.**
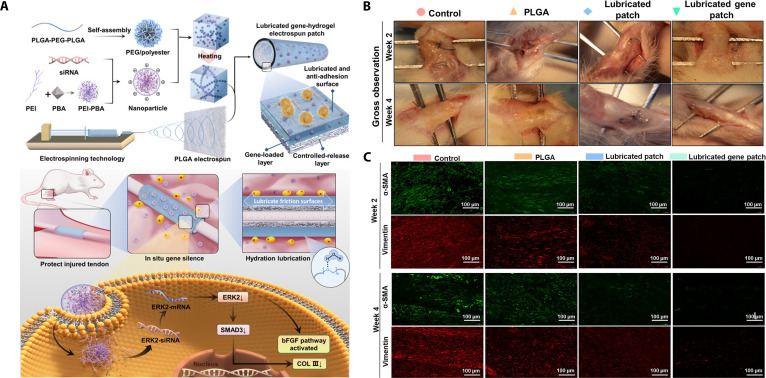
Design and fabrication of hydrogel patches for tendinopathy treatment. (A) Schematic illustration. (B) Evaluation of lubricated gene-hydrogel patch in vivo. (C) Fibrosis and vascularization assessment of healing tendons. Reprinted from Xiang et al. [193]. Copyright 2023, the authors. bFGF, basic fibroblast growth factor; COL III, type III collagen; ERK2, TGF-β1-extracellular signal-regulated kinase 2; PBA, phenyl-boronic acid; PEG, poly(ethylene glycol); PEI, polyethyleneimine; PLGA, poly(lactic-co-glycolic acid); α-SMA, α-smooth muscle actin.

Furthermore, a bilayer hydrogel patch, fabricated from methoxy PEG-poly(L-valine) and electrospun PLGA membranes, loaded with bFGF and ibuprofen, effectively isolates tendons from surrounding tissues, reduces inflammatory cell infiltration, and down-regulates the expression of pro-inflammatory cytokines, including IL-1β, TNF-α, and IL-6, thereby preventing tendon adhesion. Simultaneously, it promotes the organized alignment of collagen fiber, enhances Col I expression, and facilitates tendon regeneration [192]. Notably, Xiang et al. [193] developed a lubricated, gene-based bilayer patch (Fig. 3A) using phenylboronic acid, polyethyleneimine, TGF-β1-extracellular signal-regulated kinase 2 (ERK2), small interfering RNA (siRNA), PLGA fiber membranes, and polyethylene glycol-polyester as raw materials. The outer polyethylene glycol-polyester hydrogel establishes a motion-lubricating layer through hydrogen bonding with water molecules while inhibiting fibroblast proliferation via in situ gene silencing, thereby reducing adhesion formation and peritendinous fibrosis (Fig. 3B). This motion-lubricating layer effectively blocks ERK2-mediated activation of the TGF-β1–ERK2–SMAD3 axis, which drives tendon fibrosis and vascularization (Fig. 3C). In terms of clinical applications, VersaWrap Tendon Protector, approved by the Food and Drug Administration for tendon repair, forms a gelatinous membrane between the tendon and surrounding soft tissues. By isolating the healing tendon from adjacent structures to reduce friction and improve gliding resistance, it effectively limits the formation of tendon sheath adhesions [194].

### Hydrogel spray

Sprayable hydrogels constitute a localized drug delivery strategy wherein a device is employed to spray liquid hydrogel onto wound surfaces, forming hydrogel films via in situ crosslinking under external conditions. These hydrogel sprays offer portability, rapid in situ action, quick application, tight tissue contact, and flexible drug delivery. Yang et al. [195] developed an inflammation-responsive hydrogel spray composed of methacrylated HA and curcumin (Cur)-loaded zeolitic imidazolate framework-8 (ZIF-8)@ceric oxide nanoparticles, which facilitates large-area spraying and rapid in situ photopolymerization at tendon injury sites. The spray modulates the inflammatory response by down-regulating NF-κB and IL-17 signaling pathways, suppressing macrophage-derived inflammatory cytokines and chemokines while restoring macrophage-mediated immunoregulatory homeostasis. Concurrently, it prevents aberrant osteogenic differentiation of stem cells, thereby inhibiting heterotopic ossification and promoting appropriate tendon regeneration.

### Hydrogel MSs

**Fig. 4.**
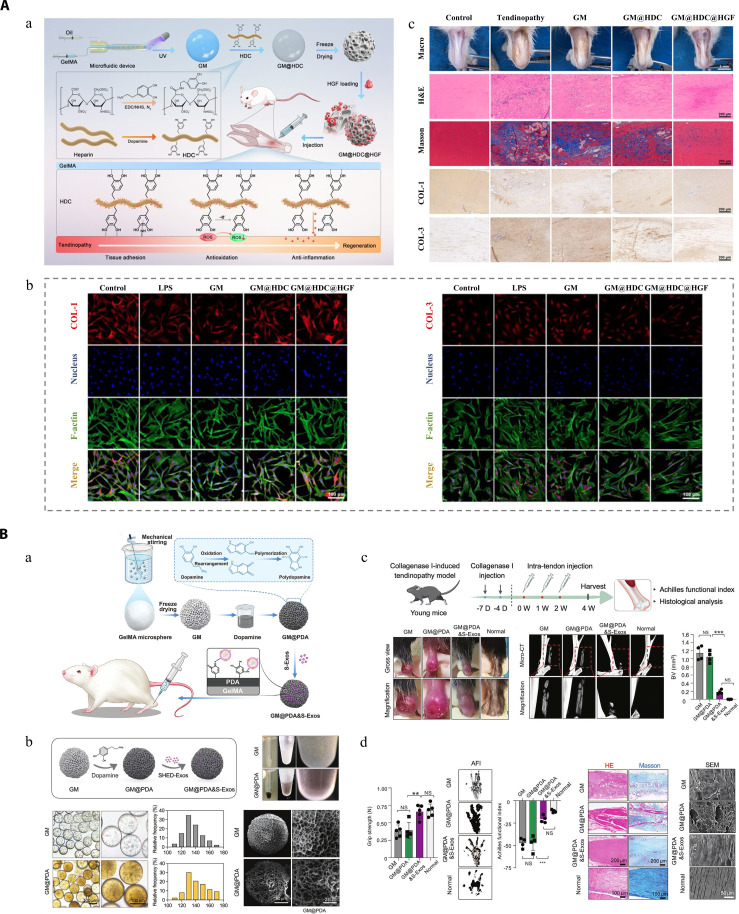
Design and fabrication of hydrogel MSs for tendinopathy treatment. (A) GM@HDC@HGF treatment of Achilles tendinopathy in rats. (a) Fabrication route of GM@HDC@HGF and molecular mechanism. (b) In vitro anti-inflammation property of MSs. (c) In vivo at regeneration with MSs treatment. Reprinted from Han et al. [199]. Copyright 2024, the authors. (B) GM@PDA&SHED-Exo micro–nano composite for tendon injury therapy. (a) Schematic illustration. (b) Characterization of GM@PDA&SHED-Exo micro–nano composites. (c) Gross view and CT scan of the tendon after local drug administration. D, days; W, weeks. (d) Evaluation of grip strength, tendon staining, and SEM in mice after local drug administration. Reprinted from Jin et al. [201]. Copyright 2023, the authors. AFI, Achilles functional index; COL-1, type I collagen; COL-3, type III collagen; EDC, N-(3-dimethylaminopropyl)-N′-ethylcarbodiimide; GelMA, gelatin methacrylate; GM, gelatin methacryloyl microspheres; HDC, heparin–dopamine conjugate; HGF, hepatocyte growth factor; LPS, lipopolysaccharide; PDA, polydopamine; ROS, reactive oxygen species; S-EXOs, young exosomes secreted by stem cells from human exfoliated deciduous teeth.

Hydrogel MSs, typically ranging in particle size from 1 to 1,000 μm, have emerged as advanced biomaterials for delivering drugs, growth factors, and stem cells in tissue repair and regeneration. These MSs demonstrate excellent biocompatibility, biodegradability, and biosafety. Additionally, the MSs can effectively adapt to irregular tendon wounds, closely conform to the wound surfaces, and enhance localized expression levels of loaded drugs at the injury site [196–198]. Cai et al. [198] designed a siRNA@MS@HA hydrogel electrospun membrane using HA and GelMA-based MSs (GMs) as raw materials. MSs can be degraded by MMP-2, enabling the on-demand release of encapsulated siRNA nanoparticles. This targeted release silences the Smad3 gene, suppressing Col I and Col III production, inhibiting fibroblast proliferation, and reducing fibrotic tissue formation to achieve anti-adhesion effects. Additionally, this system decreases macrophage infiltration while promoting polarization toward M2 phenotypes, alleviating inflammatory responses. Notably, an innovative injectable GM system loaded with a heparin–dopamine conjugate (HDC) and hepatocyte growth factor (HGF) (GM@HDC@HGF) has been developed, combining antioxidant and anti-inflammatory properties for in situ treatment of tendinopathy (Fig. 4A, a). GM@HDC@HGF down-regulates the expression of pro-inflammatory cytokines, including IL-1β, TNF-α, and IL-6, in lipopolysaccharide-stimulated tenocytes, while improving the balance of ECM-related proteins, specifically Col I and Col III (Fig. 4A, b). These effects were further substantiated in a rat model of Achilles tendinopathy (Fig. 4A, c). Regarding its antioxidant activity, GM@HDC@HGF markedly reduces ROS levels in H_2_O_2_-induced cells and up-regulates expression of the antioxidant regulator nuclear factor erythroid 2-related factor 2 and its downstream proteins, Heme oxygenase-1 and NADPH quinone oxidoreductase 1 [199].

Notably, a GelMA hydrogel system integrated with SF MSs was engineered for stem cell delivery and controlled loading and release of 2-[(aminocarbonyl)amino]-5-(4-fluorophenyl)-3-thiophenecarboxamide. This platform attenuates the production of inflammatory factors by inhibiting the IκB kinase β/NF-κB signaling pathway, thereby mitigating inflammatory responses. Furthermore, it facilitates tenogenic differentiation of stem cells at the injury site while suppressing ectopic chondrogenesis and ossification [200]. Similarly, young exosomes (Exos) secreted by stem cells from human exfoliated deciduous teeth (SHED-Exos)-loaded GM@PDA&SHED-Exos hydrogel system developed by Jin et al. [201] demonstrated multiple therapeutic benefits (Fig. 4B, a and b). It reduced senescence markers (p21 and γ-H2AX) and oxidative stress in aged TSPCs, while enhancing cellular proliferation and stemness, such as increasing the expression of octamer-binding transcription factor 4 and SRY (sex determining region Y)-box 2. Moreover, by suppressing the NF-κB signaling pathway and down-regulating inflammatory genes (Il-6 and Ccl5), the system effectively alleviates inflammation-induced functional impairment, reduces ectopic bone formation, and promotes tendon repair (Fig. 4B, c and d).

Ren et al. [202] developed a PH/GMs@bFGF&PDA hydrogel with a sophisticated design. The GMs facilitated fibroblast migration and tendon healing in the murine model through controlled loading and sustained release of bFGF. In a rabbit tendon injury model, the hydrogel demonstrated excellent adhesive properties by maintaining close contact with the injured tendon and synchronizing with its motion during physiological activity. The GMs exhibit a uniformly porous architecture, wherein electrostatic interactions between the acidic gelatin and bFGF facilitate uniform drug distribution. Furthermore, sustained release of bFGF attenuates inflammatory factor levels, accelerates inflammation resolution, and enhances cell proliferation, migration, and angiogenesis, thereby promoting tendon regeneration.

### Injectable hydrogels

Injectable hydrogels have demonstrated considerable potential as materials for in situ applications, including tendon injuries, due to their ability to control morphology and replicate the 3D microenvironment of natural tissue. The internal spatial structure of these hydrogels provides a niche for migration and differentiation of TSCs [203]. Furthermore, they can be directly injected into the injured site, eliminating the need for surgical implantation and minimizing associated tissue damage. Injectable hydrogels offer distinct advantages, including minimal invasiveness at target sites, elimination of off-target effects, reduced drug dosage requirements, and prolonged drug retention for sustained release [204].

In terms of anti-inflammatory and antioxidant stress effects, a local injectable poly(organophosphazene)-celecoxib (CXB) nanoparticle (PCNP) hydrogel system was developed to treat tendonitis. The PCNP remains in a solution at 4 °C, providing excellent fluidity that facilitates injection operations. It stays localized in the injured Achilles tendon area without dispersing as the body temperature induces a transition to a stable gel state. The PCNP system continuously releases CXB, inhibiting the expression of pro-inflammatory factors such as COX-2. Additionally, it promotes the expression of anti-inflammatory factors such as IL-4 and IL-10, thereby reducing the inflammatory response and regulating the immune microenvironment [205]. Similarly, Zhang et al. [206] developed an injectable hydrogel loaded with gastrodin, utilizing small intestinal submucosa to achieve efficient gastrodin encapsulation and controlled release. This system attenuated inflammatory responses by inhibiting the NF-κB signaling pathway, suppressing pro-inflammatory cytokine IL-6 and up-regulating anti-inflammatory IL-10 expression. With excellent biocompatibility, the hydrogel notably enhanced the proliferation and migration of tenocytes. Furthermore, it promoted tendon regeneration by enhancing Col I production and simultaneously reducing Col III expression, thereby facilitating the restoration of well-organized collagen fiber alignment. The HA hydrogel demonstrates rapid in situ gelation upon injection, ensuring precise localization at injured tendon sites to prevent drug diffusion while maintaining optimal therapeutic concentrations. The epigallocatechin gallate-loaded HA hydrogel possesses dual anti-inflammatory and antioxidant properties that effectively mitigate cellular oxidative stress damage. Furthermore, it suppresses the elevated Col III/Col I gene expression ratio in cyclically stretched TDCs, thereby minimizing fibrotic scar tissue formation [207].

**Fig. 5.**
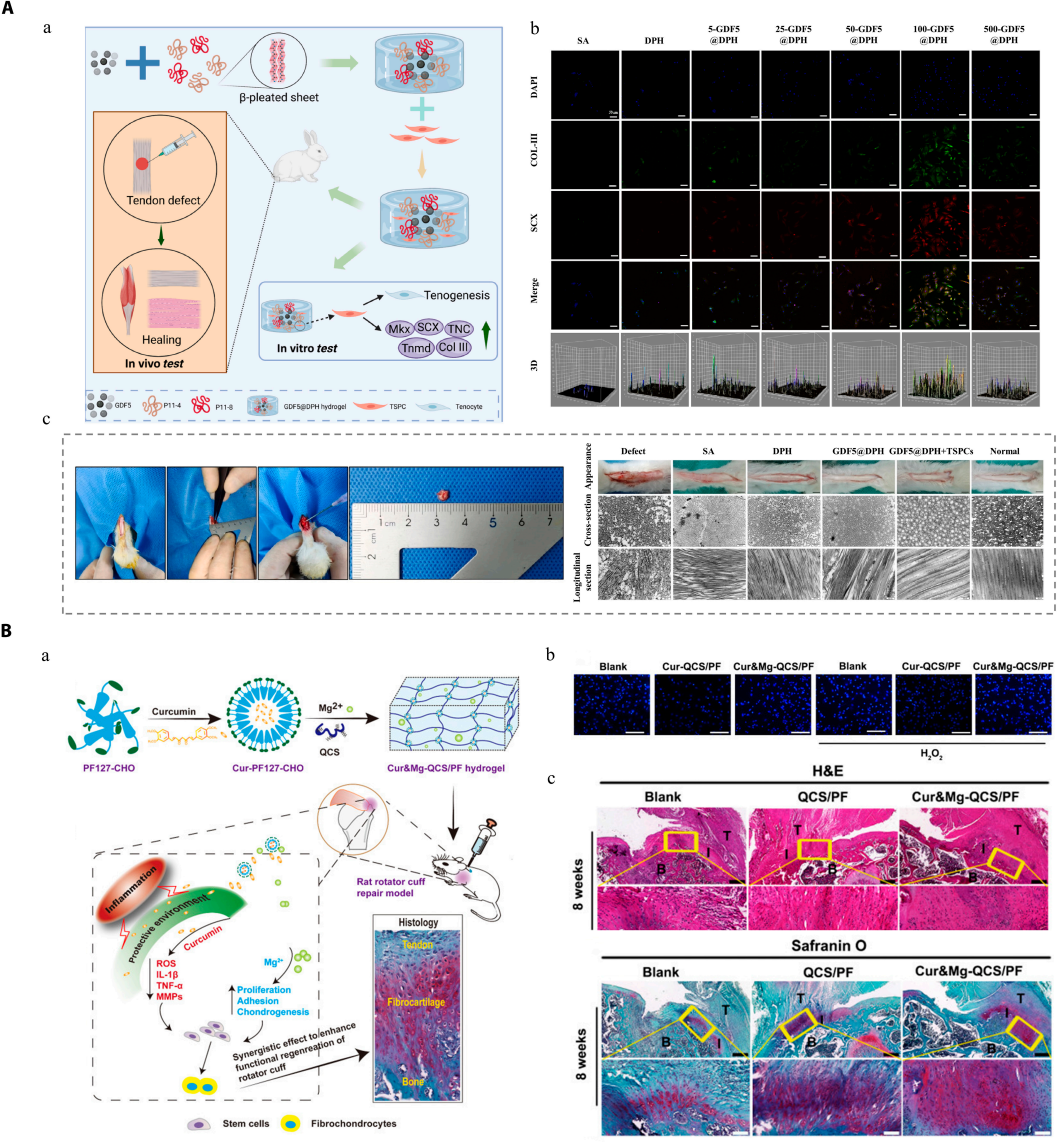
Design and fabrication of injectable hydrogels for tendinopathy treatment. (A) Injectable GDF5@DPH for tendon injury therapy. (a) Schematic illustration. (b) GDF5@DPH promotes tenogenic differentiation of TSPCs in vitro. (c) GDF5@DPH and GDF5@DPH loaded with exogenous TSPCs enhance tendon regeneration and repair in vivo. Reprinted from Zhang et al. [208]. Copyright 2024, the authors. (B) Cur&Mg-QCS/PF promotes rotator cuff healing. (a) Fabrication route of Cur&Mg-QCS/PF and molecular mechanism. (b) In vitro anti-apoptosis and antioxidant effects of curcumin-loaded hydrogels. (c) In situ regeneration of a native-like fibrocartilaginous interface between rotator cuff tendon and bone by Cur&Mg-QCS/PF. Reprinted from Chen et al. [215]. Copyright 2021, the authors. COL-III, collagen III; DAPI, 4′,6-diamidino-2-phenylindole; DPH, dipeptide hydrogel; GDF5, growth differentiation factor 5; IL-1β, interleukin-1β; MKX, mohawk homeobox; Tnmd, tenomodulin; MMPs, matrix metallopeptidases; PF127-CHO, PluronicF127; QCS, quaternized chitosan; SA, sodium alginate; SCX, scleraxis; TNC, tenascins; TNF-α, tumor necrosis factor-α; TSPCs, tendon stem/progenitor cells.

Regarding TSCs regulation, a dipeptide hydrogel (DPH) was developed using peptides P11-4 and P11-8 and subsequently incorporated with growth differentiation factor 5 (GDF5) to form an injectable GDF5@DPH system (Fig. 5A, a). When rabbit TSPCs were cocultured with GDF5@DPH, notable improvements were observed in cell viability, proliferation, and the expression of tenogenic markers, including tendon-related proteins (SCX and MKX) and genes (Scx, Tnc, Mkx, and Tnmd), indicating enhanced tenogenic differentiation of TSPCs (Fig. 5A, b). In vivo studies confirmed that the synergistic effects of GDF5 release and exogenous TSPC application increased stem cell recruitment to injured sites and improved tendon repair outcomes (Fig. 5A, c) [208]. An injectable hydrogel network encapsulating BMSC-derived Exos (BMSCs-Exos) was engineered to facilitate sustained release at injury sites. This delivery system enhanced the migration, proliferation, and tenogenic differentiation of TSPCs at injury sites, thereby accelerating tissue regeneration [209]. Fitzgerald et al. [210] employed a collagen/ALG gel-based biodegradable scaffold to achieve controlled delivery of GDF5/PDGF-primed ADSCs to tendon defect sites. Pretreatment with GDF5 and PDGF increased the proliferative capacity and tenogenic differentiation potential of ADSCs. In a tendon injury model, administration of this composite hydrogel markedly up-regulated the expression of tendon-specific markers, including TNMD, Col I, hypoxia-inducible factor 1-alpha (HIF-1α), and ROS modulator 1. The treatment enhanced collagen fiber alignment while contributing simultaneously to tendon repair and angiogenesis. Recently, a piezoelectric injectable anti-adhesion hydrogel has been reported, which can form a physical barrier in situ at the tendon injury site to reduce inflammatory responses and prevent tendon adhesion. Meanwhile, the piezoelectric short fibers incorporated within the hydrogel, when activated by ultrasound, promote the proliferation and differentiation of TSCs due to the piezoelectric effect [211].

Advancements have been achieved in integrating anti-inflammatory strategies and stem cell regulatory mechanisms. Dou et al. [212] confirmed that injectable hydrogels prepared from CS, human umbilical vein endothelial cell-derived Exos, and hydroxyethyl cellulose effectively enhanced the proliferation of TDSCs, promoted the polarization of macrophages toward the M2 phenotype, and concurrently suppressed the polarization of M1. These combined effects contributed to attenuating inflammatory responses and facilitated tissue regeneration. The injectable hydrogel, designed for easy administration to the target site via a syringe and rapid in situ gelation, enables the continuous release of oxotremorine M and 4-phenyl-1-(4-phenylbutyl) piperidine, thereby promoting the differentiation of TSCs into tendon cells. Meanwhile, it inhibits the polarization of M1 macrophages (reduces the mRNA expression of TNF-α and IL-1β) and enhances the polarization of M2 macrophages (increases the expression of mannose receptor C-type 1 and PDGFb) [213]. Wan et al. [204] developed an injectable hydrogel that promotes the proliferation and differentiation of TDSCs while inhibiting cell apoptosis and ROS production. Furthermore, this hydrogel regulates the inflammatory response by modulating NF-κB and MAPK signaling pathways, suppressing the polarization of pro-inflammatory M1 macrophages, and enhancing the polarization of anti-inflammatory M2 macrophages. Notably, the tendon-derived decellularized ECM (tdECM) hydrogel loaded with TDSCs-Exos exhibited excellent injectability before crosslinking and achieved optimal viscosity after crosslinking. The tdECM hydrogel regulates macrophage polarization via the phosphatidylinositol 3-kinase-protein kinase B and MAPK signaling pathways, mitigating inflammatory responses and exhibiting immunomodulatory properties. Besides, it regulates the proliferation, migration, and fibrogenesis of TDSCs [214]. Chen et al. [215] developed a novel injectable composite self-healing hydrogel, Cur&Mg-quaternized CS (QCS)/PluronicF-127 (PF), based on QCS and aldehyde groups from benzaldehyde-terminated PF-127 polymer (Fig. 5B, a). This hydrogel precisely controls the delivery of Cur and magnesium ion (Mg^2+^). Cur&Mg-QCS/PF facilitates the localized release of Cur and establishes a protective environment for stem cells and tendon tissues by inhibiting ROS and inflammatory cytokines (Fig. 5B, b). Furthermore, the release of Mg^2+^ from the hydrogel enhances stem cell adhesion, migration, aggregation, and cartilage differentiation. Animal experiments further demonstrated that the synergistic effect of Cur and Mg^2+^ accelerated the regeneration of new fibrocartilage tissue and organized collagen fibers, thereby enhancing biomechanical properties and promoting rotator cuff healing (Fig. 5B, c).

Research has demonstrated that activation of the Ras-related C3 botulinum toxin substrate 1 (Rac1) signaling pathway promotes the differentiation of TSPCs toward osteochondral rather than tenogenic lineages, ultimately resulting in pathological tendon calcification. A hydrogel formulated from CS, β-glycerophosphate ester, and Rac1 inhibitor NSC23766 remains liquid at room temperature, allowing for ease of injection, and rapidly transitions into a solid gel at body temperature. This system enables the in situ fixation of drugs. The release efficiency of NSC23766 can be modulated by adjusting the concentrations of CS and β-glycerophosphate. By inhibiting the Rac1 signaling pathway, NSC23766 suppresses pathological osteogenic differentiation of TSPCs while simultaneously promoting their tenogenic differentiation [216]. Administration of RADA hydrogel [Ac-(RADA)_4_-CONH_2_] loaded with TSPCs in a rat patellar tendon injury model yielded regenerated tissue exhibiting morphological characteristics closely resembling native tendon. This regenerated tissue exhibited enhanced collagen fiber organization, lower cellular density, reduced vascularization, decreased adipocyte accumulation, and suppressed ectopic ossification, collectively accelerating functional tendon repair [217].

The porous microstructure and biodegradable properties of injectable DNA hydrogels facilitate the encapsulation, preservation of bioactivity, and sustained release of cytokines [218]. Branched DNA nanostructures are programmable, orderly, and possess well-defined local domains, which facilitates the formation of functionalized DNA hydrogels [219]. Ge et al. [220] encapsulated TSPCs in a DNA hydrogel (TSPCs-Gel), where the rigidity of the DNA scaffold and the flexibility of the supramolecular DNA network protected the stem cells from external compression and shear stress. This approach prolonged TSPCs retention and promoted in vivo healing of tendon injuries in rats. Furthermore, a delivery system based on DNA supramolecular hydrogel has also been developed. Local injection of this system extended the retention time of 2-amino-6-[2-(cyclopropylmethoxy)-6-hydroxyphenyl]-4-(4-piperidinyl)-3-pyridine carbonitrile and greatly reduced the frequency of administration. It down-regulated the expression of inflammation-related genes and ECM remodeling-related genes, while up-regulating markers of tenogenic differentiation, thereby effectively mitigating inflammation and promoting tendon differentiation [221].

Injectable hydrogels have also achieved progress in clinical research and application. After repair of Zone II flexor tendons, ADCON-T/N was administered to inhibit fibroblast migration, thereby preventing scar tissue formation. This resulted in improved range of motion in the proximal interphalangeal joint of the patients [222]. Postoperative ultrasound-guided injection of carboxymethyl CS hydrogel serves as a physical barrier, providing enhanced anti-adhesion effects and improved prognosis, while also reducing pain in the short term after surgery [223]. In addition, following tenolysis of flexor tendons, the use of Hyaloglide resulted in a faster improvement in the range of motion of the affected limb and reduced the recurrence of adhesions [224]. Indeed, injectable hydrogels show great promise in the treatment of tendinopathy.

## Biomedical Trends in Stimuli-Responsive Biomaterials for Tendon Repair with Emphasis on Hydrogel-Based Formulations

Physically responsive hydrogels can detect changes in physical parameters such as temperature, pressure, and light and adjust their structure or functionality in response to these external stimuli. Chemically responsive hydrogels exhibit sensitivity to specific chemical agents, including pH, ROS, and glucose, thereby enabling regulation of their internal network structure through the breaking or formation of chemical bonds. Biologically responsive hydrogels can recognize and bind specific biomolecules, such as enzymes or antibodies, and initiate targeted responses via biological recognition mechanisms [225].

### Physical responsiveness

Stimuli-responsive hydrogels regulate drug release in response to changes within the internal environment, thereby promoting tissue repair [226]. For example, a motion-responsive hydrogel with reversible disulfide bond cleavage and recombination exhibits reversible sol-gel transition characteristics. This system maintains a protective gel state under static conditions and transitions into a lubricating sol state when subjected to mechanical stress during movement. This hydrogel mitigates the inflammatory response by exhibiting protein resistance and suppressing the TGF-β1-p-Smad2/3 signaling pathway. It also inhibits excessive Col III deposition during scar formation while promoting the expression of Col I, thereby facilitating high-quality tendon healing without adhesion formation [227].

Temperature-responsive hydrogels undergo volumetric changes in response to temperature fluctuations. These variations modify the interactions between the hydrogel matrix and the surrounding solution, thereby weakening or strengthening hydrophobic interactions within the network, which ultimately induces volumetric changes. Temperature-responsive hydrogels are classified into negative and positive temperature-sensitive hydrogels [228]. Li et al. [229] designed and synthesized a temperature-responsive hydrogel that co-delivers Mg^2+^ and BMP-12 to promote tendon–bone interface regeneration. The hydrogel undergoes rapid sol-gel transition at physiological temperature, facilitating immediate in situ gelation at the injured tendon–bone interface. BMP-12 activates the Smad signaling pathway, up-regulating tendon-related gene expression and enhancing tendon tissue regeneration. Simultaneously, the rapid release of free Mg^2+^ induces M2 macrophage polarization, suppressing early inflammatory responses and promoting osteogenic and tenogenic differentiation of BMSCs. The sustained release of Mg^2+^ mediated by hydrogel also preserves a long-term anti-inflammatory microenvironment. This staged immunomodulatory mechanism addresses distinct regenerative needs during the various tendon healing phases. Similarly, another in situ-forming temperature-responsive hydrogel remains in a liquid state at low temperatures, facilitating easy injection into the tendon repair cavity. Upon reaching body temperature, the hydrogel transitions to a gel state with minimal volume shrinkage, ensuring tight adhesion to the damaged tissue and forming a stable physical barrier that effectively blocks fibroblast invasion. Furthermore, the hydrogel’s high-water content provides excellent lubrication in vivo, reducing friction between the tendon and surrounding tissues and minimizing postoperative peritendinous adhesion [230].

Regarding magnetic responsiveness, a biomimetic tendon tissue engineering scaffold was developed by incorporating magnetic nanoparticles and Col I. Under exposure to a magnetic field, magnetic nanoparticles assemble into chain structures within the collagen hydrogel, guiding the anisotropic alignment of collagen fiber and forming an anisotropic hydrogel. This aligned architecture provides topological cues for human adipose stem cells (hASCs), facilitating their parallel alignment with the fibrous collagen structure and the orientation of magnetic nanoparticles while promoting tenogenic differentiation of hASCs through the up-regulated expression of TNMD and SCX proteins [231]. Similarly, an anisotropic, gelatin-based multiphase hydrogel system was engineered by incorporating cellulose nanocrystals aligned under an external magnetic field. Within this anisotropic hydrogel, hASCs exhibited parallel orientation of their longitudinal axes with the alignment of cellulose nanocrystals. Moreover, the secretion of tendon-associated matrix proteins, such as TNC, demonstrated ordered deposition patterns aligned with cellular orientation. This coordinated cell alignment and directional ECM deposition potentially facilitate structural regeneration and functional restoration of tendon tissue [232].

### Chemical responsiveness

Using ZIF-8 as an imatinib mesylate (IM) carrier, a pH-responsive hydrogel, IM@ZIF-8@Gel, was developed to treat tendon adhesion. The IM@ZIF-8@Gel system facilitates the sustained release of IM under acidic conditions, which simulate the microenvironment following a tendon injury, extending the drug’s activity and providing higher drug concentrations during the critical period of adhesion formation. The released IM effectively inhibited myofibroblast proliferation and adhesion, substantially reducing expression of Col III, p-ERK1/2, p-signal transducer and activator of transcription 3 (STAT3), and Claudin 1 (CLDN1) to suppress fibrosis while exerting minimal impact on TSCs. In a rat Achilles tendon adhesion model, IM@ZIF-8@Gel decreased Col III and CLDN1 expression in adhesion tissues, effectively preventing adhesion formation without adversely affecting tendon healing [233].

### Biochemical responsiveness

Chen et al. [234] synthesized an MMP-2-responsive hydrogel by crosslinking GelMA with MMP-2 substrate peptides and incorporating it with IL-4. Subsequently, a core–shell structure was formed through layer-by-layer CS/HA/stromal cell-derived factor-1 (SDF-1) electrostatic deposition onto the core particles. In response to elevated MMP-2 levels at the tendon injury site, enzymatic degradation of MMP-2 substrate peptides within the GelMA network triggered hydrogel dissociation. The released IL-4 activated the Janus kinase 3/STAT6 signaling pathway, promoting macrophage polarization toward the M2 phenotype to exert anti-inflammatory responses and facilitate tissue repair. Simultaneously, the initial rapid release of SDF-1 recruited MSCs to the injury site, establishing a cellular foundation for subsequent cartilage regeneration and tissue repair.

### Multiresponsiveness

To address the hypoxic, acidic, redox, and enzyme-rich microenvironment at the tendon–bone interface, a multiresponsive hybrid interpenetrating 3D network hydrogel was designed and fabricated. Boronate ester bonds facilitated the pH-responsive release of deferoxamine (DFO) at the acidic inflammatory microenvironment of injury sites. The disulfide bonds embedded within the hydrogel maintain stability under oxidative conditions but undergo cleavage in reductive environments, enabling on-demand drug release. The hydrogel’s enzyme-responsive properties also achieve localized and adaptive drug delivery in response to microenvironmental changes. The controlled release of DFO up-regulates HIF-1α, enhancing the expression of VEGF, BMP-2, and Runx2, which collectively promote angiogenesis and osteogenesis. Moreover, this system effectively mitigates oxidative stress by inducing macrophage polarization toward the M2 phenotype, alleviating inflammation and accelerating tissue repair. In an anterior cruciate ligament reconstruction model, this approach strengthened tendon graft-to-bone integration, facilitated interfacial healing, and promoted the formation of a native fibrocartilage-resembling transitional structure [235].

## Concluding Remarks and Future Perspectives

We have summarized the application progress of hydrogels in tendinopathy based on their formulations and responsiveness. Special emphasis is placed on the efficacy of distinct hydrogel formulations (e.g., injectable, scaffolds, and patches) in promoting tendon tissue regeneration through structural and biochemical modulation. Hydrogels have emerged as an exciting and highly promising approach for tendon tissue engineering and repair. The superior biocompatibility and bioactivity of hydrogels render them a potentially ideal therapeutic strategy for tissue repair. Hydrogels in various formulations hold great promise, but their application in tendon regeneration requires further exploration to achieve clinical translation as early as possible. In the future, hydrogel design may incorporate comprehensive evaluations of injury site, severity, and individual variability to enable more precise and intelligent personalized therapies.

However, hydrogels still possess several limitations that hinder their practical applications. Due to their high-water content, hydrogels exhibit poor mechanical properties. Since tendons are load-bearing tissues with high strength and toughness, conventional hydrogels remain insufficiently robust to mimic or replace natural human tendons. High loads and deformation in practical applications can compromise the structural integrity of hydrogels, leading to undesirable degradation and unstable release of drugs or other therapeutic agents. The underlying mechanisms by which hydrogels promote tendon repair remain poorly understood. Tendon tissue regeneration during repair and the formation of fibrosis or adhesions are regulated by multiple cell populations (e.g., stem cells and macrophages). Due to insufficient knowledge about the roles of different cell types in tendon repair, current treatment approaches rarely achieve ideal clinical outcomes. Although various attempts have been made to design functionally adaptive hydrogels to modulate cell behavior for tendon repair, the mechanisms behind the tendon repair process and cell-biomaterial interactions remain elusive [236].

The surgical procedures for tendon repair are relatively cumbersome, as implanted scaffolds must be sutured or otherwise attached to tendon tissue. This prolongs surgical time and increases the risk of postoperative complications. Moreover, due to variations in tendon injury types (e.g., flexor tendons, Achilles tendon, or rotator cuff tendons), locations (e.g., the tendon itself or the bone–tendon interface), and patient conditions (e.g., diseases or age), a range of grafts with different structures and functions should be developed to meet each patient’s anatomical, histological, biochemical, and biomechanical requirements. The methods, techniques, dosage, and treatment duration for hydrogel injection require further optimization. Current research remains limited to cellular and animal experiments, primarily in rodents. Key challenges include transitioning to primate studies, safely and effectively advancing preclinical and clinical research, establishing unified clinical application standards, and achieving successful clinical translation. Gaps remain before practical clinical application can be realized.

The advancement of artificial intelligence (AI) technology has brought new opportunities to hydrogel research, offering advantages in hydrogel design and optimization such as performance prediction and enhancement, and high-throughput screening, while also promoting hydrogel applications across various biomedical fields. AI and machine learning will assist in optimizing material selection and composition. These technologies will help establish accurate predictive models, thereby improving research and development efficiency and providing deeper insights into modified performance variations. AI-assisted high-throughput screening can optimize 3D printing parameters and comprehensively cover all aspects of hydrogel composition screening, material preparation, and performance optimization. By analyzing extensive medical data and domain-specific knowledge bases, AI systems can rapidly identify optimal material formulations and accurately predict material properties through simulation and computational modeling. Furthermore, machine learning algorithms enable intelligent process optimization to enhance the standardization of hydrogel fabrication, ensuring product consistency while achieving breakthrough improvements in production efficiency. Moreover, models can be constructed to predict drug release behavior, algorithms can be optimized to enhance drug sustained-release system performance, and image processing/recognition technologies can monitor drug release. AI holds advantages in data analysis and simulation prediction, enabling AI-assisted prediction of hydrogel crosslinking to improve hydrogel stability and optimize hydrogel design [237–239].

## Data Availability

Data sharing is not applicable for this article as no datasets were generated or analyzed during the current study.
